# Efficacy, safety, and tolerability of antimicrobial agents for complicated intra-abdominal infection: a systematic review and network meta-analysis

**DOI:** 10.1186/s12879-023-08209-9

**Published:** 2023-04-21

**Authors:** Wenqiang Kong, Ting Deng, Shiqin Li, Yunfeng Shu, Yanyan Wu

**Affiliations:** 1 Department of Pharmacy, Zi Gong First People’s Hospital, Zi Gong, China; 2 Department of Pharmacy, Women and Children’s Hospital of Chongqing Medical University, Chongqing, China

**Keywords:** Complicated intra-abdominal infection, Antimicrobial agents, Systematic review, Network meta-analysis, Efficacy, Safety, Tolerability

## Abstract

**Background:**

Which antimicrobial agents provide the optimal efficacy, safety, and tolerability for the empirical treatment of complicated intra-abdominal infection (cIAI) remains unclear but is paramount in the context of evolving antimicrobial resistance. Therefore, updated meta-analyses on this issue are warranted.

**Methods:**

We systematically searched four major electronic databases from their inception through October 2022. Randomized controlled trials examining antimicrobial agents for cIAI treatment were included. Two reviewers independently assessed the quality of included studies utilizing the Cochrane Collaboration’s risk of bias tool as described in the updated version 1 of the Cochrane Collaboration Handbook and extracted data from all manuscripts according to a predetermined list of topics. All meta-analyses were conducted using R software. The primary outcome was clinical success rate in patients with cIAIs.

**Results:**

Forty-five active-controlled trials with low to medium methodological quality and involving 14,267 adults with cIAIs were included in the network meta-analyses. The vast majority of patients with an acute physiology and chronic health evaluation II score < 10 had low risk of treatment failure or death. Twenty-one regimens were investigated. In the network meta-analyses, cefepime plus metronidazole was more effective than tigecycline and ceftolozane/tazobactam plus metronidazole (odds ratio [OR] = 1.96, 95% credibility interval [CrI] 1.05 ~ 3.79; OR = 3.09, 95% CrI 1.02 ~ 9.79, respectively). No statistically significant differences were found among antimicrobial agents regarding microbiological success rates. Cefepime plus metronidazole had lower risk of all-cause mortality than tigecycline (OR = 0.22, 95% CrI 0.05 ~ 0.85). Statistically significant trends were observed favoring cefotaxime plus metronidazole, which exhibited fewer discontinuations because of adverse events (AEs) when compared with eravacycline, meropenem and ceftolozane/tazobactam plus metronidazole (OR = 0.0, 95% CrI 0.0 ~ 0.8; OR = 0.0, 95% CrI 0.0 ~ 0.7; OR = 0.0, 95% CrI 0.0 ~ 0.64, respectively). Compared with tigecycline, eravacycline was associated with fewer discontinuations because of AEs (OR = 0.17, 95% CrI 0.03 ~ 0.81). Compared with meropenem, ceftazidime/avibactam plus metronidazole had a higher rate of discontinuation due to AEs (OR = 2.09, 95% CrI 1.0 ~ 4.41). In pairwise meta-analyses, compared with ceftriaxone plus metronidazole, ertapenem and moxifloxacin (one trial, OR = 1.93, 95% CI 1.06 ~ 3.50; one trial, OR = 4.24, 95% CI 1.18 ~ 15.28, respectively) were associated with significantly increased risks of serious AEs. Compared with imipenem/cilastatin, tigecycline (four trials, OR = 1.57, 95%CI 1.07 ~ 2.32) was associated with a significantly increased risk of serious AEs. According to the surface under the cumulative ranking curve, Cefepime plus metronidazole was more likely to be optimal among all treatments in terms of efficacy and safety, tigecycline was more likely to be worst regimen in terms of tolerability, and eravacycline was more likely to be best tolerated.

**Conclusion:**

This study suggests that cefepime plus metronidazole is optimal for empirical treatment of patients with cIAIs and that tigecycline should be prescribed cautiously considering the safety and tolerability concerns. However, it should be noted that data currently available on the effectiveness, safety, and tolerability of antimicrobial agents pertain mostly to lower-risk patients with cIAIs.

**Supplementary Information:**

The online version contains supplementary material available at 10.1186/s12879-023-08209-9.

## Background

Intra-abdominal infections (IAIs) are a common problem in clinical practice and pose a major challenge for clinicians due to high morbidity and mortality rates. Traditionally, IAIs are classified as uncomplicated intra-abdominal infections and complicated intra-abdominal infections (cIAIs) [1]. cIAIs are defined as infections that extend into a normally sterile area of the abdomen, such as gastroduodenal perforations, and commonly required a source control procedure [1–3]. Successful management of these infections relies on timely and appropriate source procedures and appropriate empirical antimicrobial therapy. Source control procedures (e.g., percutaneous drainage) play a fundamental role by removing infected intra-abdominal fluid and tissue [1]. Empiric antimicrobial therapy, albeit as a supplement to source control, is important in the overall treatment plan. Compared with proper selection of empiric antibiotics, inappropriate empiric antibiotic therapy is associated with higher risk of clinical failure, increased hospital length of stay, and higher medical costs [4, 5], thus highlighting the importance of selecting the optimal empirical therapy regimen.

cIAIs tend to be polymicrobial infections, which are generally caused by Gram-negative Enterobacteriaceae, Gram-positive cocci, and obligate anaerobes [1]. Of these micro-organisms, *Escherichia coli* or *Klebsiella pneumoniae, Streptococci* and *Bacteroides fragilis* are predominantly isolated from intra-abdominal fluid and tissue cultures. The wide variety of microorganisms causing cIAIs has resulted in the use of broad-spectrum antimicrobial agents for empirical treatment [1, 3]. Many antimicrobial agents are potential choices for treating cIAIs. In addition to older antimicrobials, several novel agents, such as eravacycline, ceftazidime/avibactam, and ceftolozane/tazobactam, has been approved for the treatment of patients with cIAIs [6, 7]. Pairwise meta-analyses [8–10] have provided limited evidence suggesting optimal regimens for empirical therapy due to limitations associated with comparing only two interventions at a time. Given changes in patient’ characteristics and resistance over time in conjunction with the large number of antimicrobials available, proper selection of an empiric antimicrobial agent for cIAIs is challenging. In addition, inappropriate or overuse of antimicrobial therapy is associated with an increased risk of resistant pathogen emergence, higher medical costs, and even increased mortality risk. Therefore, it is important to determine the optimal empiric therapy for each cIAI and generate hierarchies of the efficacy, safety, and tolerability of available antimicrobial agents. Network meta-analyses (NMAs) that simultaneously compare all interventions of interest by statistically combining direct and indirect evidence, is probably the best approach for identifying agents exhibiting the best efficacy, safety, and tolerability for the empirical treatment of cIAIs [11].

## Methods

We conducted this network meta-analysis to compare all available drugs in the treatment of patients with cIAIs according to the specifications of the Preferred Reporting Items for Systematic Reviews and Meta-analyses (PRISMA) extension statement for reporting of systematic reviews incorporating network meta-analyses of healthcare interventions [12]. The protocol was prospectively registered at PROSPERO International Prospective Register of Systematic Reviews (https://www.crd.york.ac.uk/PROSPERO/) under the registration number CRD42022313771.

### Search strategy and study selection

Three electronic databases, PubMed, EMBASE, and the Cochrane Central Register of Controlled Trials (CENTRAL), were systematically searched from their inception to October 2022 to identify eligible randomized controlled trials (RCTs). We also searched *ClinicalTrials.gov* to identify completed but unpublished RCTs. The references list of relevant meta-analyses, reviews, pooled analyses, and included trials were manually checked to identify additional studies. We used combinations of MeSH terms and text words around “intra-abdominal infection”, “antimicrobials”, and “randomized controlled trials” (see Supplementary table S1).

Two reviewers (WQK, YYW) independently selected the studies in accordance with pre-specified inclusion criteria, and any disagreements were settled via discussion. After removing duplicate studies, the reviewers screened the titles and abstracts of the remaining records, read the full text of the remaining reports, and identified eligible studies.

### Inclusion criteria

The inclusion criteria for studies were as follow: (1) RCTs; (2) included adults 18 years of age or older with a cIAI; (3) evaluated antimicrobial agents, including fluoroquinolone monotherapy or fluoroquinolone plus metronidazole, β-lactam/β-lactamase inhibitor monotherapy or β-lactam/β-lactamase inhibitor plus metronidazole, cephalosporin monotherapy or cephalosporin plus metronidazole, or carbapenems or other antimicrobial regimens that ensured coverage of common pathogens involved in IAIs, compared with control groups receiving active agents; (4) had to report at least one clinical-related outcome (e.g., clinical success rate or microbiological success rate); (5) constituted patients with cIAIs as a subgroup of the study population, and provided separate data for the group of patients with cIAIs; (6) published in English language or Chinese language (see Supplementary table S2).

Studies that evaluated aminoglycosides were excluded due to recommendations against their use in the 2017 Surgical Infection Society revised guidelines on the management of IAI [1] and aminoglycosides-associated nephrotoxicity. Studies focusing on patients with peritoneal dialysis associated peritonitis by reasons of differences in management were also excluded, as were studies evaluating agents in the same antibiotic class (e.g., meropenem versus imipenem/cilastatin). Non-RCTs, conference abstracts, and unpublished studies were also excluded. When duplicate studies were identified, only those that presented the most comprehensive and informative data were included.

### Data extraction

Two reviewers (WQK, YYW) independently extracted data from studies that met the inclusion criteria, and the data were managed using Microsoft Excel spreadsheets. One reviewer (WQK) then performed a thorough additional check to ensure that the data were accurate. The data included study characteristics (first author, study design, ClinicalTrials.gov Identifier [NCT], publication year, sample size, therapy duration), participant characteristics (mean age, sex ratio, mean acute physiology and chronic health evaluation II [APACHE II] score); interventions (drugs and dose); concomitant antibiotics; and outcomes of interest. The primary outcome was clinical success rate, which was defined as complete resolution or significant improvement in all signs and symptoms of the infection such that no additional antibiotic or procedure was necessary at the test-of-cure visit or the end-of-therapy visit. Secondary outcomes included microbiological success rate defined as eradication or presumed eradication of the baseline pathogen (if no postbaseline specimen was available for culture, microbiological outcome was based on clinical assessment), all-cause mortality, serious adverse events (SAEs), and discontinuation due to adverse events (AEs). As a result of the lack of a standard definition of SAE, we used the criteria as defined in each study. We preferentially extracted a modified intention-to-treat (ITT) population for clinical success, if possible. Otherwise, we used ITT population or available data (i.e., clinically evaluable population). For microbiological success, we extracted a microbiologically modified ITT population, or used the ITT population or available data (i.e., microbiologically evaluable population). The safety outcome was evaluated in the safety population. The total number of events and sample size were extracted for all outcomes. Discrepancies were resolved via discussion.

### Risk of bias assessment

The quality of the included trials was assessed in accordance with the Cochrane Collaboration’s risk of bias tool, as described in updated version 1 of the Cochrane Collaboration Handbook [13]. Two investigators (WQK, YYW) independently determined the risk of bias to be low, unclear, or high based on the presence or absence of random sequence generation (selection bias), allocation concealment (selection bias), blinding of participants and personnel (performance bias), blinding of outcome assessors (detection bias), incomplete outcome data (attrition bias), selective reporting (reporting bias), or “other source of bias” (other bias). Discrepancies were resolved via discussion.

### Data analysis

Data were analyzed using a stepwise approach. Pairwise meta-analyses were performed first. The outcomes were dichotomous data, and results are presented as pooled odds ratios (ORs) with 95% confidence intervals (CIs). A random-effects model was used to derive pooled estimates across studies such models account for between-study differences. Between-study heterogeneity was quantitatively assessed using the *I*^2^ statistic, with *I*^2^ > 50% indicating high heterogeneity and *I*^2^ < 50% indicating low heterogeneity[14]. A Bayesian network meta-analysis (NMA) was then conducted using a random-effects model to combine direct and indirect comparisons via Markov chain Monte Carlo methods with the GeMTC package (version 0.8-2) in R (version 4.1.3) to calculate ORs and 95% credibility intervals (CrIs) [15, 16]. Four Markov chains were run simultaneously with 50 000 simulated draws after a burn-in of 20 000 iterations. Convergence of the model was assessed using trace plots and the Brooks-Gelman-Rubin statistic [17]. Consistency was evaluated using node-splitting analysis comparing the differences between direct and indirect estimates for each comparison [18, 19]. Model fit was assessed by comparing the deviance information criteria (DIC) between the consistency model and inconsistency model, which indicated a good fit if the number of DIC was approximated [20]. If data were available, ORs and 95% CrIs for the primary outcome were calculated separately on the basis of APACHE II scores ≥ 10. In order to validate the robustness of findings for all outcomes, a series of four sensitivity analyses were performed to: [1] exclude trovafloxacin, which has been withdrawn due to serious hepatotoxicity; [2] remove studies at high risk of blinding bias; [3] remove studies with an enrolled sample size of ≤ 100 patients; [4] exclude studies with missing information regarding the APACHE II score. Furthermore, a network meta-regression analysis was conducted to explore the impact of study publication year on all outcomes. The surface under the cumulative ranking (SUCRA) curve and mean ranks were calculated to rank the treatments for each outcome [21]. The Stata software version 14.0 was utilized to draw network plots [22]. To quantitatively evaluate the possible impact of publication bias in the NMA, Egger’s tests were conducted on all the outcomes [23].

## Results

### Selected studies and study characteristics

**Fig. 1:**
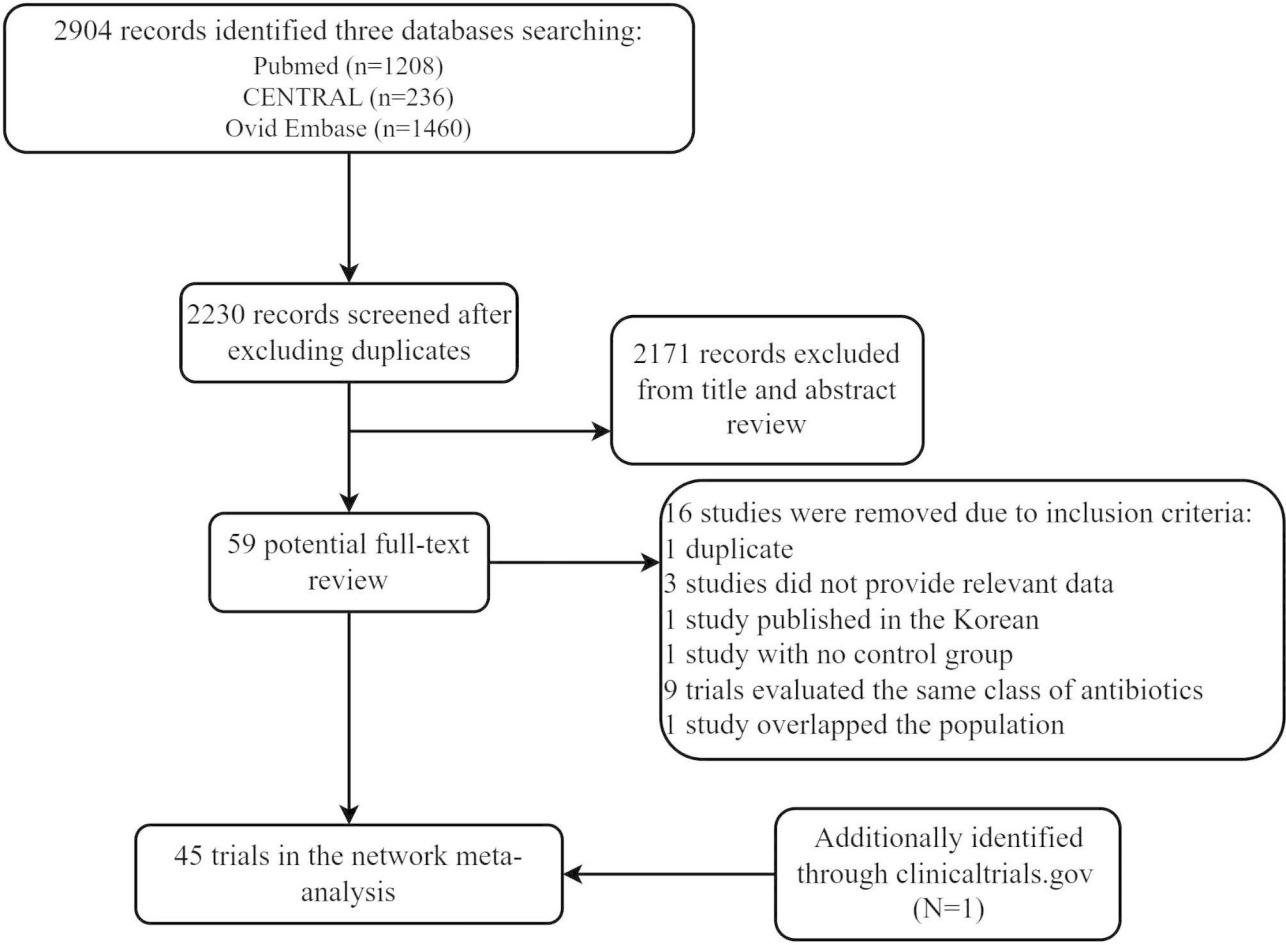
Flow diagram of study selection

The literature screening process is shown in Fig. 1. Electronic searches yielded 2,904 citations, and the full-text versions of 59 publications were subsequently reviewed. Of these, 16 publications were rejected based on the inclusion criteria. Forty-three studies comprising 44 trials met the inclusion criteria and were included in the network meta-analysis. One trial from *ClinicalTrials.gov* [24] that had completed results but had not been published met the criteria and was included. Forty-five English-language RCTs published between 1992 and 2021 were ultimately included in the NMA. Basic information derived from each of the 45 RCTs is shown in (see Supplementary table S3). All studies were active-controlled trials. Twenty-one anti-infection regimens were investigated in the NMA (see Supplementary box 1). A total of 38.5% (5,499/14,267) of the included subjects were female. The sample size varied from 56 to 1,043. Twenty-eight RCTs were multicenter studies with a double-blind design. The vast majority of patients had an APACHE II score ≤ 10, and thus considered at low risk for treatment failure or death [1]. Nine trials did not report any data on disease severity. The mean age of included patients ranged from 35 to 66 years. The study duration ranged from 4 days to 14 days. Vancomycin, teicoplanin, linezolid, or daptomycin were concomitantly used against resistant gram-positive pathogens in 10 studies. Thirty-seven RCTs disclosed financial support from the pharmaceutical industry.

### Quality of the included studies

Detailed random sequence generation was not reported in 20 of the included studies (44.4%), and 23 studies (51.1%) had inadequate allocation concealment. Fourteen trials (31.1%) were open-label design, 20 (44.4%) studies did not describe how participants and personnel were blind, and 23 (51.1%) studies were rated as having an unclear risk of outcome assessment blinding. Thirty-one (68.9%) trials were rated as having low-risk bias in terms of incomplete outcome data. Seventeen (37.8%) studies were considered to have high-risk bias on the basis of selective reporting. Thirty-five (77.8%) trials were deemed to have low-risk of other bias. (See Supplementary table S4)

### Assessment of convergence, mode fit and consistency

Trace plots and Brooks-Gelman-Rubin plots indicated good model convergence in the NMAs (see Supplementary figure S1A ~ S1D). The consistency and inconsistency models exhibited good fit according to approximated DIC (See Supplementary table S5). No significant inconsistencies were noted between direct and indirect evidence, as assessed using the node-splitting method (see Supplementary table S6).

### Clinical success rates

Results for clinical success rates were obtained from 45 RCTs covering 21 treatment regimens and involving 16,646 participants The network plot of the outcome appeared in Fig. 2A. The clinical success rate was 82.6% (13,750/16,646). In the NMA, cefepime plus metronidazole (CEP_M) was more effective than tigecycline (TGC) and ceftolozane/tazobactam (TT_M) int terms of clinical success rate, although the differences were of borderline statistical significance (OR = 1.96, 95% CrI 1.05 ~ 3.79; OR = 3.09, 95% CrI 1.02 ~ 9.97, respectively) as shown in Fig. 3A. Sensitivity meta-analyses confirmed the robustness of the CEP_M against TGC (see Supplementary league table S1A ~ S1D). Ertapenem (ERM) was more effective than TGC in terms of clinical success rate when studies with small sample sizes were excluded (OR = 1.54, 95% CrI 1.04 ~ 2.39) or studies at high risk of blinding bias (OR = 1.54, 95% CrI 1.03 ~ 2.38) The results of pairwise meta-analyses were consistent with those of the NMAs (see Supplementary table S7). According to SUCRA curve analysis, CEP_M had the highest probability (91.2%) of being the best regimen among all treatments in terms of clinical success rate (see Supplementary table S8), whereas cefotaxime plus metronidazole (COX_M) had the lowest probability (12.0%). Network meta-regression analysis suggested that publication year had no potentially moderating association with treatment(see Supplementary table S9). Twelve of the included articles provided data relative to APACHE II scores ≥ 10 for clinical success rates, totaling nine treatment arms, including ceftazidime/avibactam plus metronidazole (CA_M), meropenem (MEM), TT_M, ERM, piperacillin/tazobactam (PT), COX_M, moxifloxacin (MOF), ceftriaxone plus metronidazole (CTX_M), and TGC. The NMA results showed that none of the antimicrobial regimens were significantly superior, however, CrIs around ORs were very wide (see Supplementary league table S1).

**Fig. 2A:**
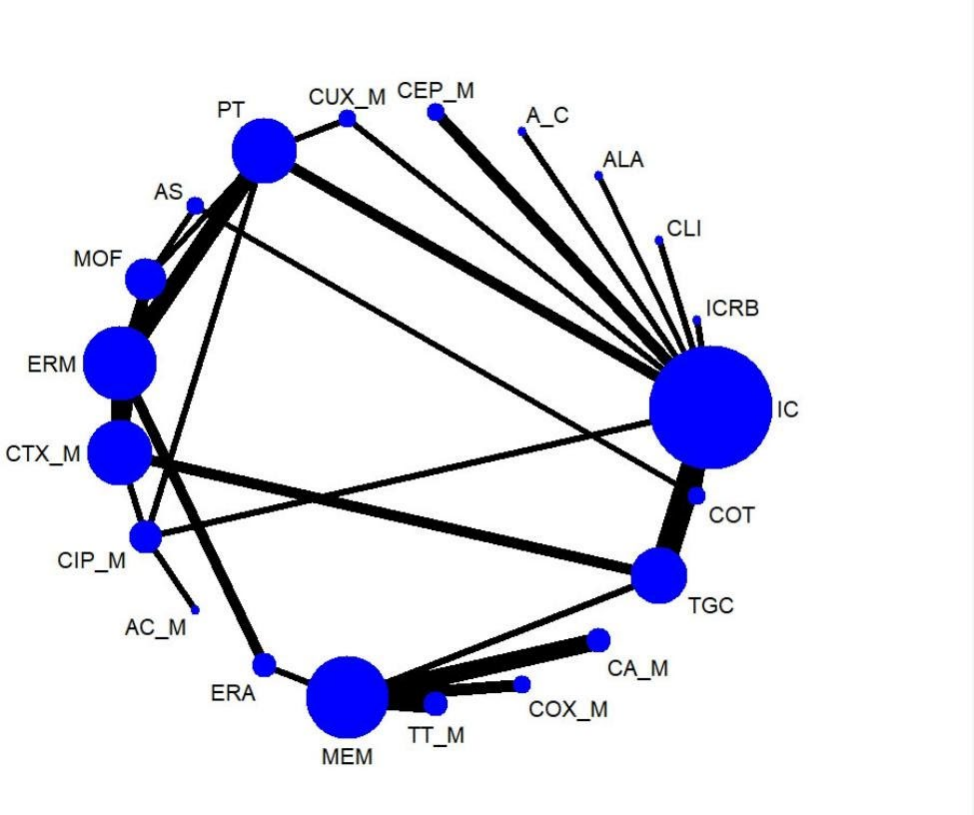
The network plot for clinical success rates

### Microbiological success rates

Thirty-one RCTs including a total of 7,733 patients reported the microbiological success rates of 15 antimicrobial treatments. However, three comparisons including MEM versus CA_M, MEM versus COX_M and MEM versus TT_M, were excluded from the NMA due to disconnections in the network. The network plot for the outcome is shown in Fig. 2B. Microbiological success was reported in approximately 85.9% of patients (6646/7733). The results of pairwise meta-analyses were generally consistent with those of NMAs (see Supplementary table S7). No statistically significant differences were observed in terms of the comparative efficacies of any of the treatment regimens in achieving microbiological success (see Fig. 3A). Several sensitivity analyses confirmed the robustness of NMA results (see Supplementary league table S2A-S2C). According to SUCRA curve analysis, CEP_M (82.8%) was the most effective in terms of microbiological success rate, and MOF (19.4%) was the least effective of the treatments examined (see Supplementary table S8). The results of network meta-regression analysis demonstrated that study publication year did not affect the outcomes (see Supplementary table S9).

**Fig. 2B:**
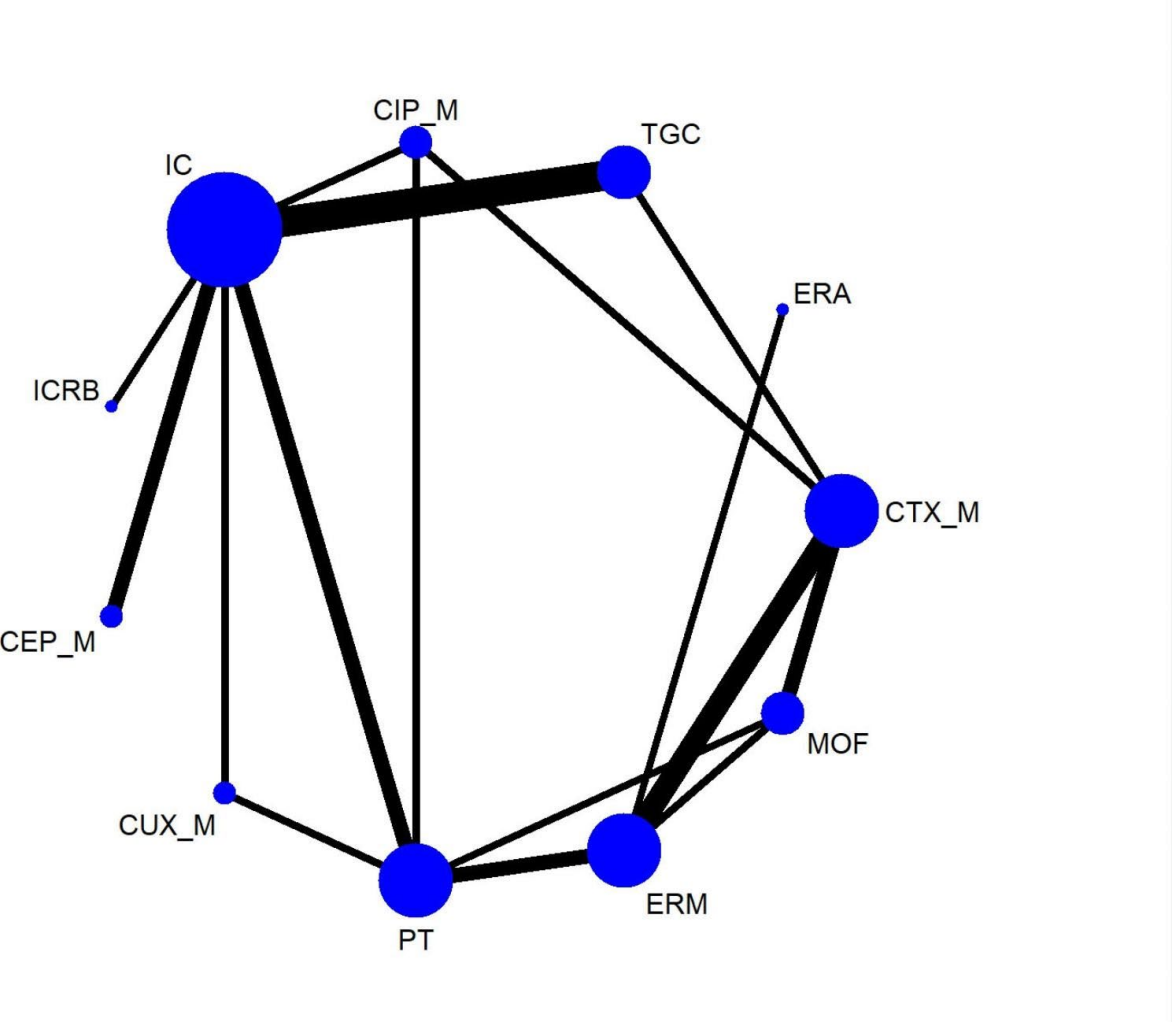
The network plot for microbiological success rates

**Fig. 2C:**
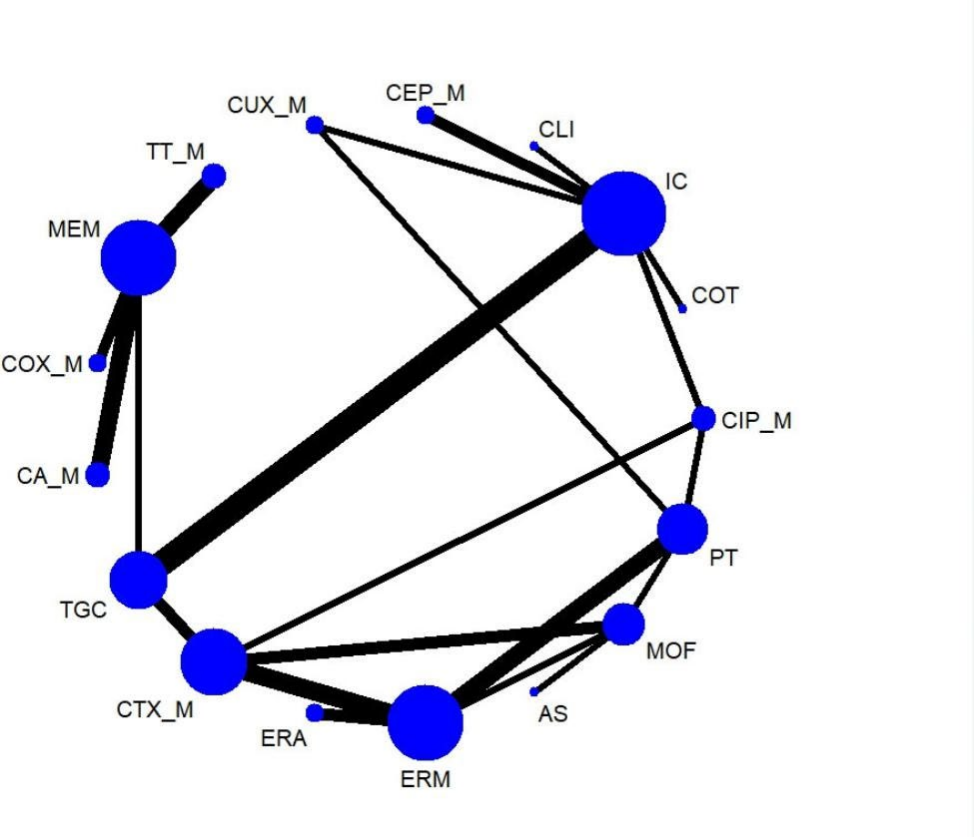
The network plot for all-cause mortality

**Fig. 2D:**
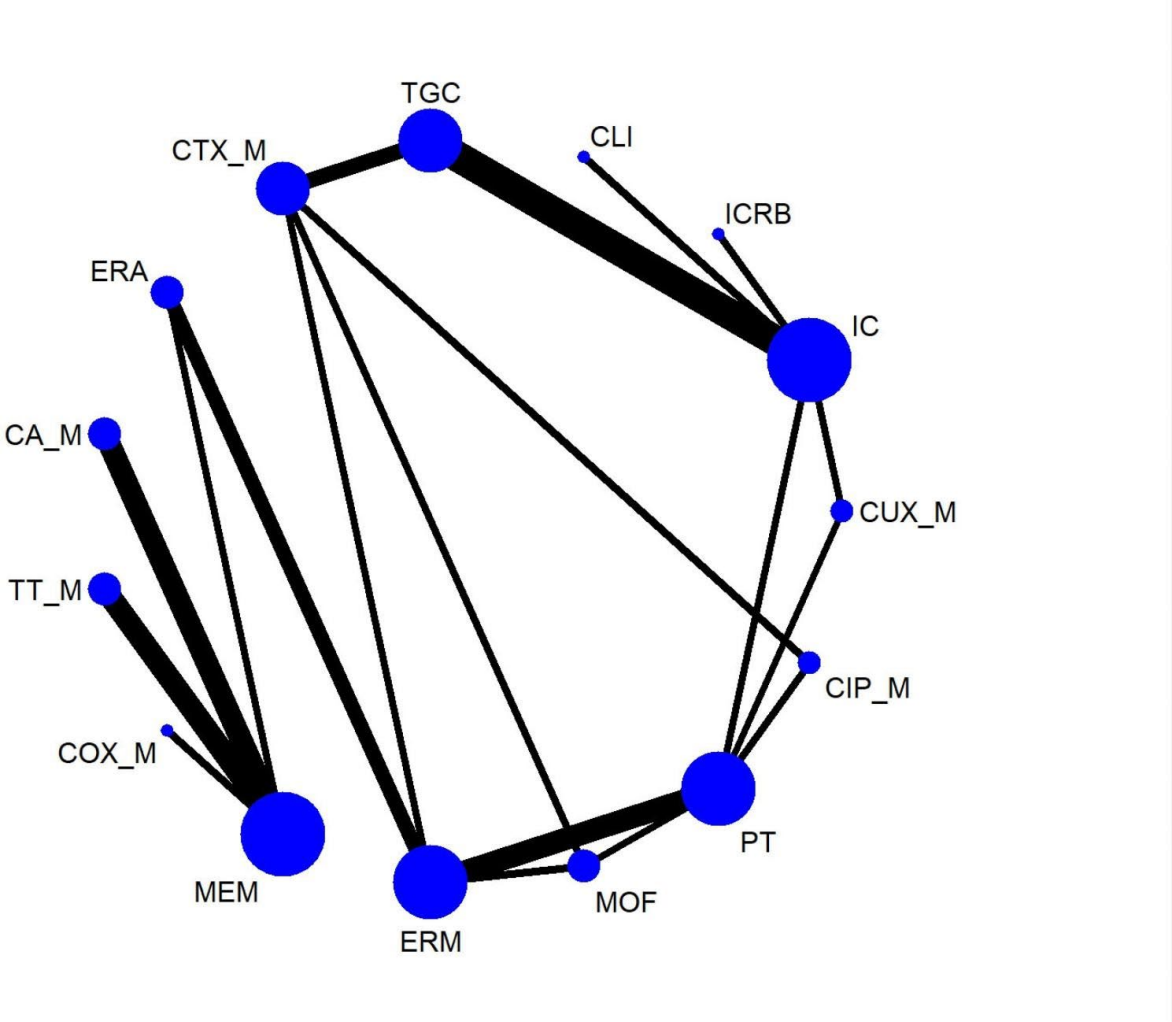
The network plot for discontinuation due to adverse events

**Fig. 3A:**
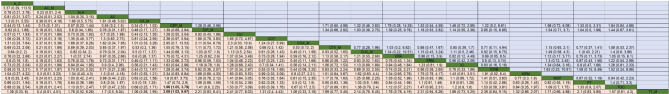
The results of network meta-analysis for clinical success rates and microbiological success rates Note: Data are OR (95% CrI) of the row treatment relative to the column treatment in terms of clinical success rates (orange) and microbiological success rates (blue). Bold values indicate comparisons that are statistically significant. ORs above 1 indicate higher rates of clinical success rates and microbiological success rates.

### All-cause mortality

Thirty-seven eligible RCTs involving 15,194 patients provided all-cause mortality data for 17 regimens. The network plot of the outcome is shown in Fig. 2C. Mortality occurred in 3.05% of patients (464/15,194). The results of pairwise and sensitivity meta-analyses were generally consistent with those of NMAs (see Supplementary table S7 and league table S3a). In the NMA, CEP_M was associated with significantly fewer cases of all-cause mortality than TGC (OR = 0.22, 95%CrI 0.05 ~ 0.85) (see Fig. 3B). According to SUCRA curve analysis, CEP_M (86%) had the lowest all-cause mortality, and CA_M (22.1%) was ranked last (see Supplementary table S8). Network meta-regression analysis revealed that publication year was not an effect modifier across trials in terms of all-cause mortality (**see Supplementary table S9**).

**Fig. 3B:**
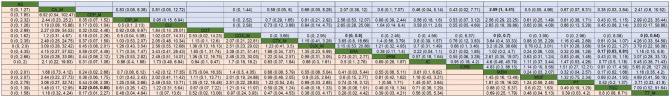
The results of network meta-analysis for all-cause mortality and discontinuation due to adverse events Note: Data are OR (95% CrI) of the row treatment relative to the column treatment in terms of all-cause mortality (orange) and discontinuation due to adverse events (blue). Bold values indicate comparisons that are statistically significant. ORs above 1 indicate higher risks of all-cause mortality and discontinuation due to adverse events.

### Serious adverse events

Twenty-five RCTs involving 11,954 participants provided serious adverse event (SAE) data for 14 interventions. SAEs were seen in 11.39% of patients (1362/11,954). Node-split analysis revealed inconsistencies in three comparisons, including PT versus imipenem/cilastatin (IC) (P = 0.02), TGC versus IC (P = 0.019), and TGC versus CTX_M (P = 0.02). As NMAs rely on an assumption of transitivity when incorporating all evidence [25], we only conducted pairwise meta-analyses. In the pairwise meta-analyses, ERM and MOF were associated with significantly more cases of SAEs than CTX_M (one trial, OR = 1.93, 95% CI 1.06 ~ 3.50; one trial, OR = 4.24, 95% CI 1.18 ~ 15.28; respectively), (see Supplementary table S7). TGC had a significantly higher rate of SAEs than IC (four trials, OR = 1.57, 95%CI 1.07 ~ 2.32).

### Discontinuation due to adverse events

Thirty-one trials covering 14,659 adults provided data for discontinuation due to adverse events (AEs). However, the comparison of ampicillin-sulbactam with cefoxitin was excluded in the NMA due to disconnected network. The network plot of the outcome is shown in Fig. 2D. A total of 3.4% of patients (496/14,659) discontinued treatment due to AEs. In the NMA, comparisons of eravacycline (ERA), MEM, and TT_M treatment with COX_M exhibited statistical significance for more patients discontinuing because of AEs (OR = 0.0, 95% CrI 0.0 ~ 0.8; OR = 0.0, 95% CrI 0.0 ~ 0.7; OR = 0.0, 95% CrI 0.0 ~ 0.64, respectively) (see Fig. 3B). ERA was associated with significantly fewer patients discontinuing because of AEs compared with TGC (OR = 0.17, 95% CrI 0.03 ~ 0.81). The comparison of CA_M with MEM exhibited borderline statistical significance for more patients discontinuing due to AEs (OR = 2.09, 95% CrI 1.0 ~ 4.41). Sensitivity and pairwise meta-analyses confirmed the robustness of the ORs of the NMAs (see Supplementary table S7 and league table S4A). According to SUCRA curve analysis, eravacycline (84.0%) exhibited the highest tolerability, whereas tigecycline (12.9%) exhibited the lowest tolerability (see Supplementary table S8). Network meta-regression analysis revealed that publication year was not an effect modifier across trials in terms of discontinuation due to AEs (see Supplementary table S9).

### Publication bias

Funnel plots of publication bias across included studies revealed generally visualized symmetry, and Egger’s test results suggested no significant publication bias among the studies included in the meta-analyses for all outcomes (Fig. 4A ~4 E).

**Fig. 4A:**
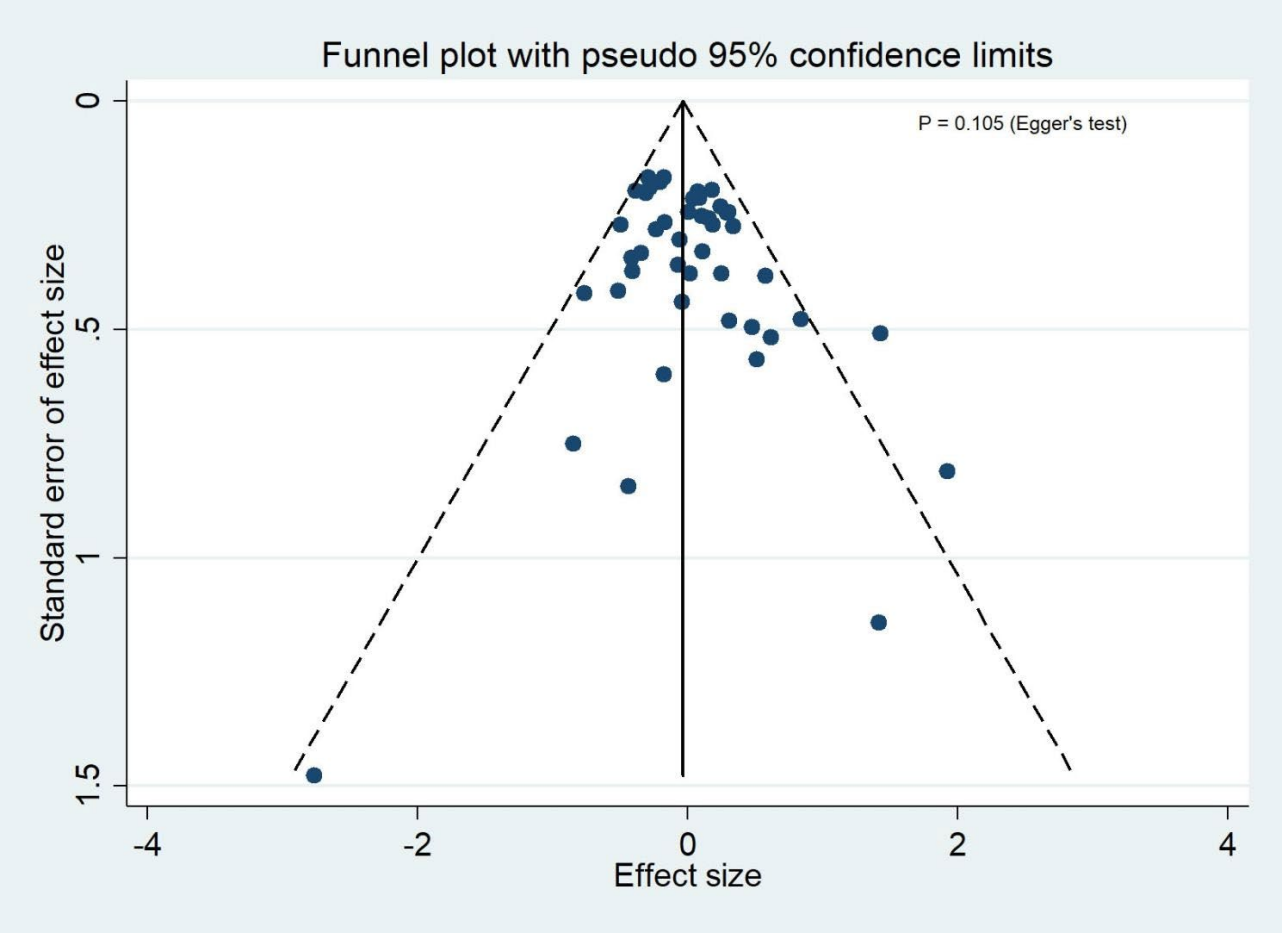
The funnel plot for clinical success rates

**Fig. 4B:**
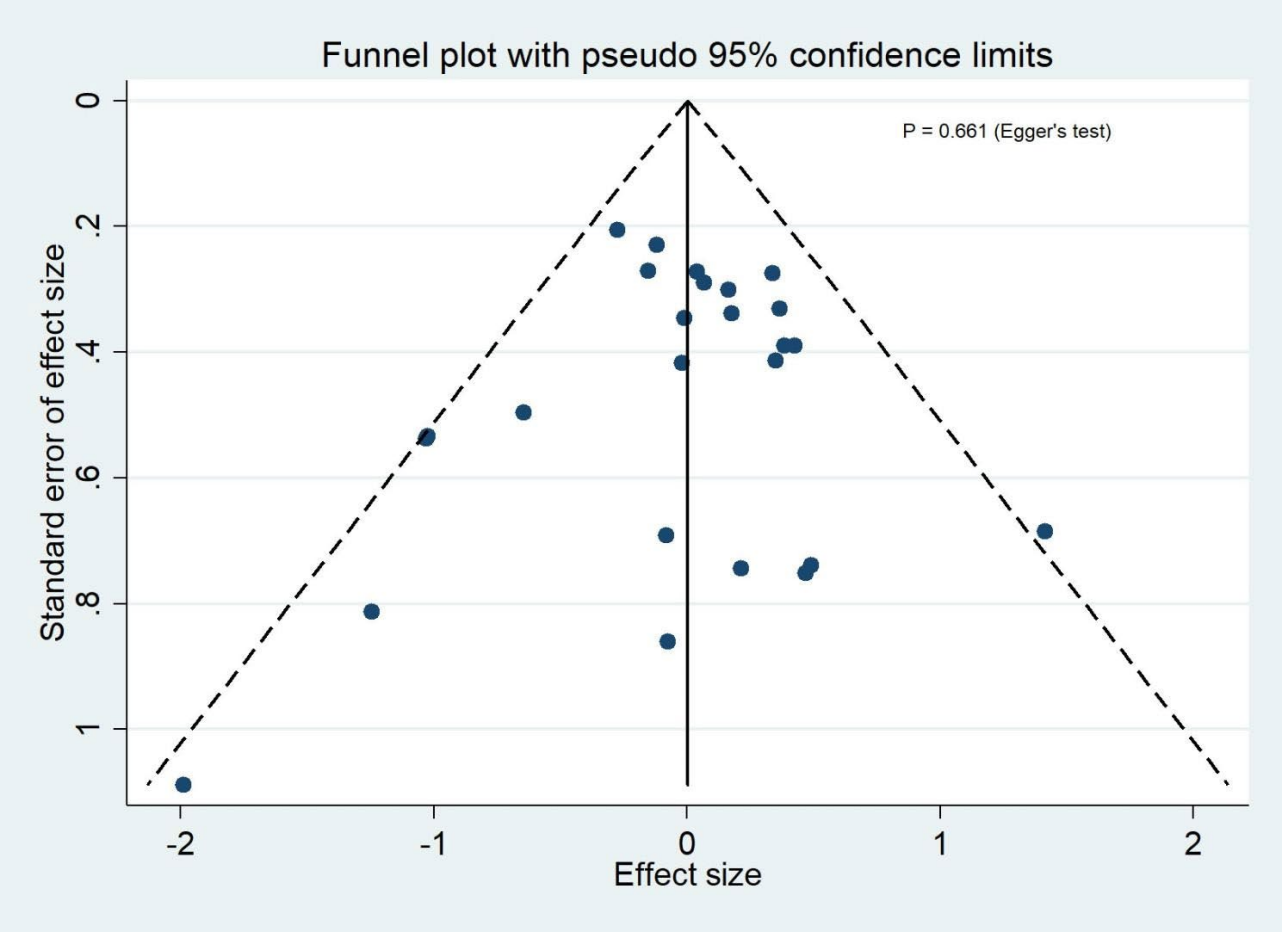
The funnel plot for microbiological success rates

**Fig. 4C:**
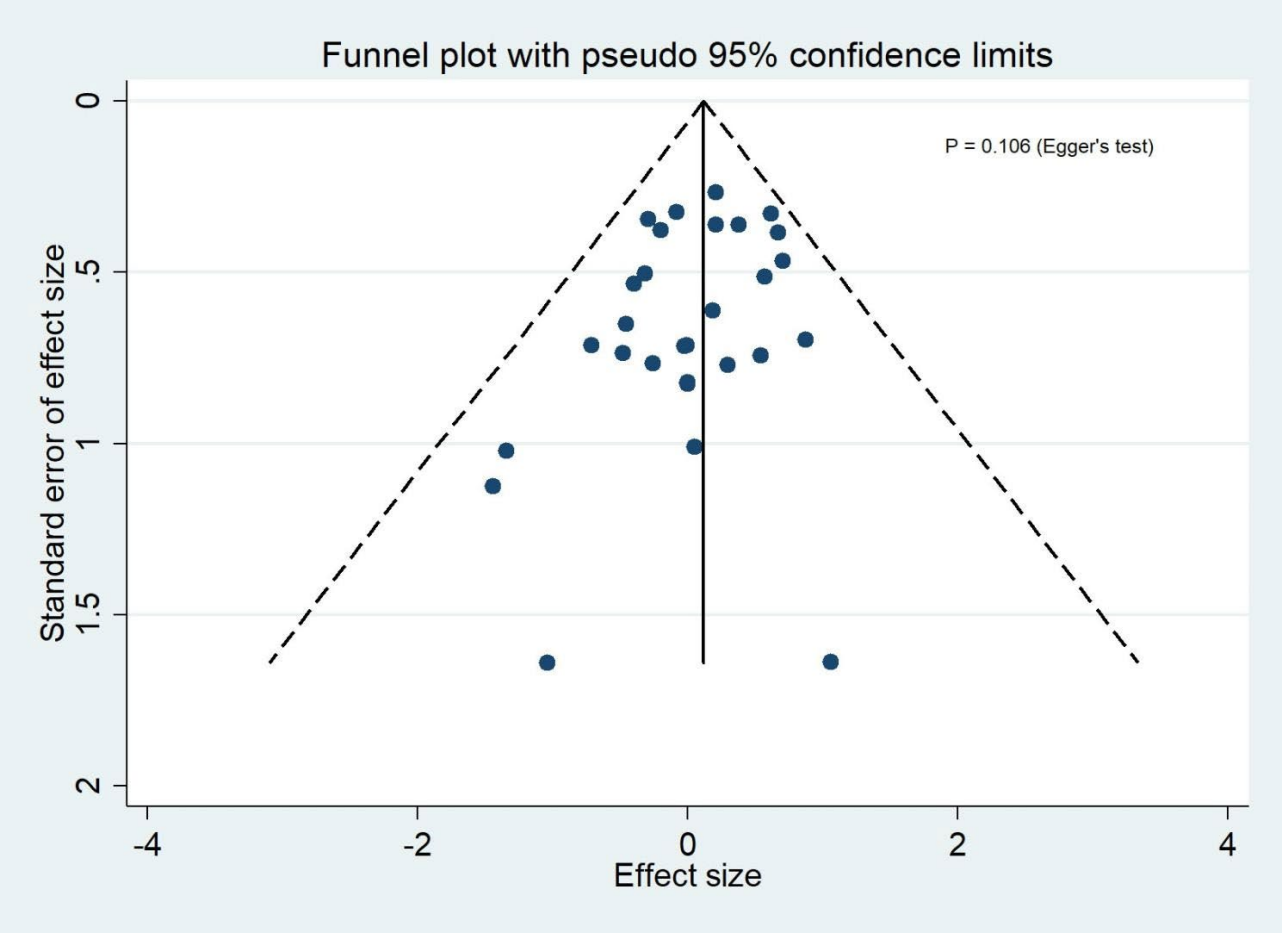
The funnel plot for all-cause mortality

**Fig. 4D:**
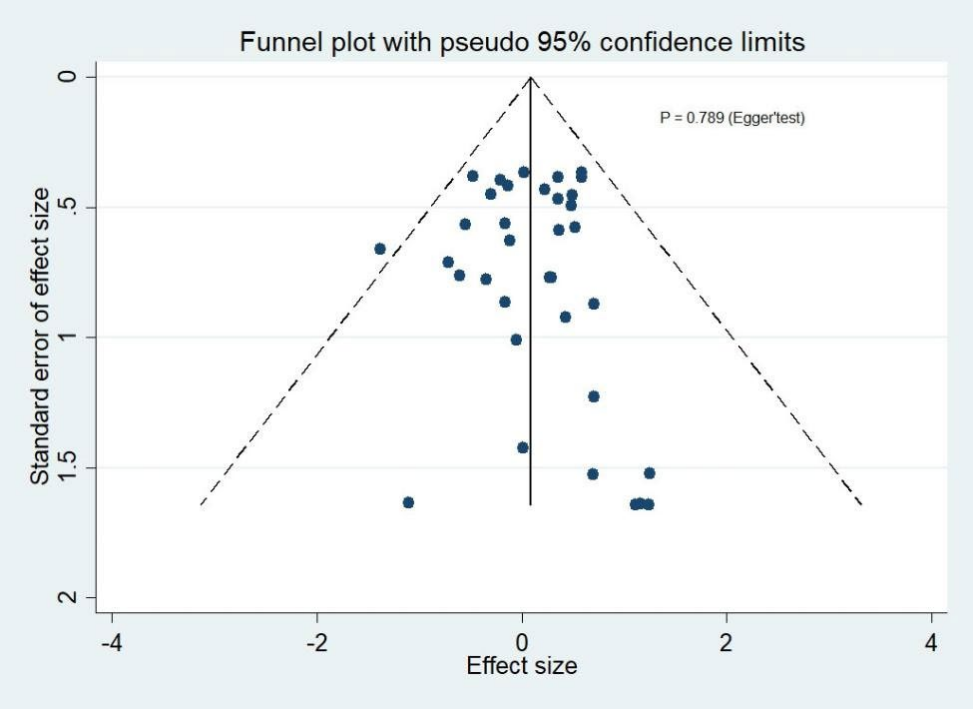
The funnel plot for serious adverse events

**Fig. 4E:**
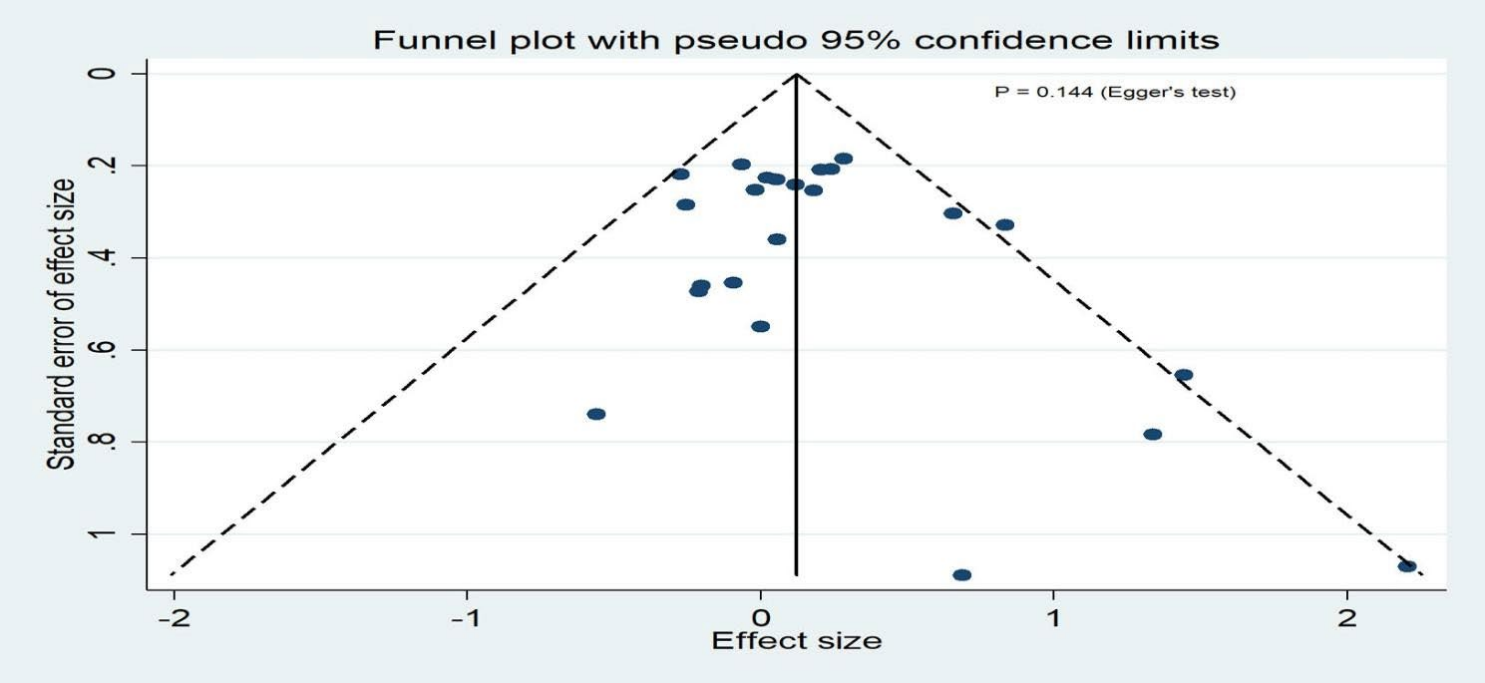
The funnel plot for discontinuation due to adverse events

## Discussion

To the best of our knowledge, the current analysis involving pooling of direct and indirect comparisons has provided the most comprehensive available evidence of comparisons of the efficacy, safety, and tolerability of various antimicrobial regimens for treating cIAIs. Our protocol was prospectively registered with PROSPERO in order to minimize the chances of duplication and reduce the possibility of reporting bias. Our NMA incorporated data on 21 antimicrobial regimens across 45 RCTs to compare differences and determine the relative ranking of antibiotics for empirical therapy for cIAIs in terms of efficacy, safety, and tolerability. Our study is timely considering the introduction of novel antimicrobials (e.g., ceftolozane/tazobactam, eravacycline) that clinicians can prescribe as a potential treatments plan for cIAIs in adults. Compared with several previous pairwise meta-analyses [8–10, 26–29], our study provided comprehensive evidence regarding the relative efficacy, safety, and tolerability of different antimicrobial regimens and clear evidence with respect to which specific antimicrobial regimens are the best candidates for the empirical treatment of cIAIs.

A major finding of our study was that CEP_M is more efficacious than tigecycline and ceftolozane/tazobactam plus metronidazole with regard to clinical success rate and appears to provide the greatest benefits among the examined therapeutic regimens in terms of clinical and bacteriological outcomes. These results did not change based on sensitivity analyses. Therefore, a cefepime plus metronidazole regimen isa superior therapeutic choice for patients with cIAIs. Few studies have explored differences in clinical efficacy among cefepime plus metronidazole, tigecycline and ceftolozan/tazobactam plus metronidazole. The present results further support the recommendations of Surgical Infection Society, whose 2017 guidelines [1], suggest the use of a cephalosporin plus metronidazole for initial empirical therapy in low-risk patients with cIAIs. Clinical data from 3233 cIAI patients published in 2016 by Ouyang et al. indicated that a cephalosporin plus metronidazole should be the first-line option for empirical therapy for cIAI patients [5], which strengthens our results. Sensitivity analysis tests in which studies with small sample sizes and those at high risk of blinding bias were excluded indicated that ertapenem is more effective than tigecycline based on clinical success rates. Our NMAs indicated that other antimicrobial regimens (monotherapy or in combination with metronidazole) provide comparable effects in terms of clinical and microbiological outcomes, in line with previous meta-analyses [8–10, 26–29], suggesting that these regimens may be suitable for initial empirical therapy in cIAI patients.

Illness severity is an important factor guiding the selection of empirical antimicrobial therapy and optimization of source control methods. A high APACHE II score is associated with an increased risk of adverse outcomes in cIAI patients. Current guidelines recommend that cIAI patients with an APACHE II score ≥ 10 be considered at increased risk of adverse outcomes in future management [1]. The present study is the first NMA to examine differences between antimicrobial agents for higher-risk cIAI patients in terms of clinical efficacy, and none of the nine treatments examined, including two novel β-lactam/β-lactamase combination antibiotics (ceftazidime/avibactam, ceftolozane/tazobactam), was optimal. Unfortunately, an insufficient number of events reported in only 12 studies increased the uncertainty of the results. Future studies should examine more data from separate studies of high-risk patients to verify our findings.

The second main finding of this study was that tigecycline was associated with an increased risk of all-cause mortality and SAEs compared with cefepime plus metronidazole and imipenem/cilastatin, respectively. Excess mortality in patients with some infections (i.e., hospital-acquired pneumonia) who received tigecycline raises considerable concern [30, 31]. Our results indicate that the use of tigecycline at the standard dose of an initial 100 mg followed by 50 mg every 12 h did contribute to increased mortality in cIAI patients when compared with cefepime plus metronidazole. Furthermore, tigecycline alone may the least tolerable, as defined by withdrawal due to AEs, among all regimens. Thus, our findings suggest that tigecycline may not be a good first choice for empirical therapy in patients with cIAIs because of its poor safety and tolerability profiles, which again confirms the current guideline[1]. These findings diverge from previous guidelines issued in 2010 that indicated the use of tigecycline for the initial empirical treatment of mild-to-moderate community-acquired infections [3]. Our findings reflect real-time and supplementary evidence regarding the efficacy and safety of tigecycline compared with other regimens for cIAI treatment. Indeed, other studies have shown higher rates of AEs in patients treated with tigecycline relative to other treatments[32, 33]. Nevertheless, tigecycline remains an important treatment option for patients suspected of being infected with an antibiotic-resistant pathogen [1, 34, 35].

Finally, considering the efficacy, safety, and tolerability profiles of novel antimicrobials for cIAIs, including eravacycline, ceftazidime/avibactam, ceftolozane/tazobactam, and imipenem/cilastatin/relebactam, our present NMA results indicated the comparability of these regimens to carbapenems or tigecycline in terms of clinical and microbiological outcomes, consistent with previous meta-analyses [26–28]. However, eravacycline was significantly better tolerated than tigecycline. Eravacycline, a fully synthetic novel fluorocycline, exhibits potent in vitro activity against Gram-positive, Gram-negative, and anaerobic bacteria, including carbapenem-resistant Enterobacteriaceae and vancomycin-resistant Enterococcus *spp.* [6]. Thus, eravacycline is a better option for the treatment of adults with cIAIs compared with tigecycline, especially as an empirical therapy for resistant pathogens. Furthermore, our NMA provided evidence that two novel beta-lactam/beta-lactamase inhibitors plus metronidazole were similar to eravacycline for empirical therapy of cIAIs with respect to efficacy, safety, and tolerability. Given the increasing prevalence of antibiotic-resistant gram-negative pathogens, especially carbapenem-resistant *K. pneumoniae* [36], our findings provide useful information to inform clinicians prescribing empirical antimicrobials for suspected infections with difficult-to-treat Gram-negative pathogens. These findings also provide evidence regarding effective carbapenem-sparing options to preserve the activity of these agents.

Several limitations of our NMA should be considered in the interpretation of our results. First, we failed to conduct an NMA for serious adverse events due to inconsistencies in some comparisons. Seven studies [37–43] used defined criteria for SAEs, whereas the remaining studies classified SAEs based on the investigators’ judgement. Discrepancies between studies in terms of judging SAEs could lead to statistical inconsistencies. Second, as with other meta-analyses, heterogeneity was an inherent limitation. Although we used strict eligibility criteria to ensure that the studies included were as homogeneous as possible, differences in participant characteristics, study duration, and study design might have increased the heterogeneity. These confounding factors may have weakened the internal validity of the evidence. However, the publications did not provide sufficient patient-level data or study characteristics, which impeded the examination of sources of heterogeneity. Third, the methodological quality of some studies was low; for example, ≥ 50% of trials did not provide adequate information about allocation concealment, and 31.1% of studies lacked blinding of participants and outcome assessors. The high risk of bias could diminish the reliability and robustness of the findings, although sensitivity analyses confirmed the consistency of the results for most comparisons. Fourth, we did not include placebo-controlled studies owing to ethical standards, which prevented assessment of the efficacy and safety of antibiotic treatment versus no antibiotic treatment. Therefore, we only synthesized outcome data for the relative efficacy and safety of agents used for the treatment of cIAIs. Including only active-controlled studies may have contributed to smaller relative effects compared with included placebo-controlled studies. The borderline significance results in our study should be interpreted conservatively [44]. Perhaps a small true difference does exist among regimens for cIAI patients, but additional high-quality data are urgently needed to clarify this issue. Fifth, although we conducted a separate NMA on the comparative efficacy of antibiotics in high- risk patients, only 12 studies with low event numbers were included. Therefore, the results are inconclusive, and more data will be needed to verify the findings. Sixth, most studies included few individuals who were more vulnerable to treatment failure or death. For example, some studies excluded patients with an APACHE II score > 30; thus, our findings should be extrapolated cautiously in this population. Seventh, as local bacterial epidemiology, pharmacokinetic and pharmacodynamic data on antibacterial agents, cost-effectiveness and availability of antibiotics, and physiopathologic factors all play a role in the selection of empirical antimicrobial therapies, it is impossible to take into account all factors in this study. For example, there is high prevalence of ESBL producing *Escherichia coli* in China, and 51% of *E. coli* are resistant to cefotaxime or ceftriaxone (http://www.chinets.com/Document/Index?pageIndex=0) and must be prescribed cautiously for cIAIs patients in China. However, about 90% of *E. coli* in North America are susceptible to cefotaxime or ceftriaxone, thus, cephalosporin-based regimens are recommended for the treatment of lower-risk patients with cIAIs [1].

In summary, as the most recent and thorough meta-analysis on this subject to date, this work has important clinical implications. All comparisons between current drugs should be considered within the context of the limitations of this NMA. We hope that our results will provide helpful perspectives to facilitate decision-making by patients and clinicians.

## Electronic supplementary material

Below is the link to the electronic supplementary material.

Supplementary Material 1

## Data Availability

All data generated or analyzed during this study are included in this published article.

## Abbreviations

TT_M: Ceftolozane/tazobactam + metronidazole
MEM: Meropenem
CLI: Clinafloxacin
IC: Imipenem/cilastatin
ERA: Eravacycline
ERM: Ertapenem
ICRB: Imipenem/cilastatin/relebactam
ALA: Alatrofloxacin
AS: Ampicillin-sulbactam
COT: Cefoxitin
PT: Piperacillin/tazobactam
CTX_M: Ceftriaxone + metronidazole
CIP_M: Ciprofloxacin + metronidazole
AC_M: Amoxicillin/clavulanic + metronidazole
COX_M: Cefotaxime + metronidazole
MOF: Moxifloxacin
CUX_M: Cefuroxime + metronidazole
TGC: Tigecycline
CEP_M: Cefepime + metronidazole
CA_M: Ceftazidime/avibactam + metronidazole
A_C: aztreonam + clindamycin
cIAI: Complicated intra-abdominal infection
RCT: Randomized controlled trial
SUCRA: Surface under the cumulative ranking curve
NMA: Network meta-analysis
APACHE II: Acute physiology and chronic health evaluation
IIDIC: Deviance information criterion
ORs: Odds ratios
PRISMA: Preferred Reporting Items for Systematic Reviews and Meta-analyses
CENTRAL: Cochrane Central Register of Controlled Trials
