# Supplementary material for: Efficacy, safety, and tolerability of antimicrobial agents for complicated intra-abdominal infection: a systematic review and network meta-analysis
Source: BMC Infect Dis. 2023 Apr 21;23:256. doi: 10.1186/s12879-023-08209-9 (PMC10122415; doi:10.1186/s12879-023-08209-9)
Supplement: Supplementary file 1 — Supplementary Material 1 [file 12879_2023_8209_MOESM1_ESM.pdf]

Supplementary Material

Supplementary table S1. Search strategies

Supplementary table S2. PICOS table

Supplementary table S3. General characteristics of studies included in the systematic review

Supplementary box 1. Antimicrobial regimens eligible for inclusion criteria in the network meta-analysis

Supplementary table S4. Risk of bias for all included studies

Supplementary table S5. Assessment of Bayesian consistency model fit for all endpoints

Supplementary table S6. Assessment of local inconsistency by node split method for all endpoints

Supplementary table S7. Network meta-analyses and pairwise meta-analyses for all endpoints

Supplementary table S8. Ranking of interventions for all endpoints by Surface Under the Cumulative Ranking (SUCRA) Curve

Supplementary table S9. Network regression for publication year

Supplementary figure S1A~S1D. The trace plot and the Brooks-Gelman-Rubin plot for all endpoints

Supplementary league table S1 Network meta-analysis results for clinical success rates based on data from patients with APACHE II score >/= 10

Supplementary league table S1A~S4A. Sensitivity meta-analysis results for all endpoints

References

Supplementary table S1 search strategies

**PubMed**	#1 Intraabdominal Infections/drug therapy"[Mesh]<br>#2 (Infection, Intraabdominal OR Infections, Intraabdominal OR Intraabdominal Infection OR Intra-Abdominal Infections OR Infection, Intra-Abdominal OR Infections, Intra-Abdominal OR Intra Abdominal Infections OR Intra-Abdominal Infection)<br>#3 #1 OR #2<br>#4 "Anti-Infective Agents/therapeutic use"[Mesh]<br>#5 (Agents, Anti-Infective OR Anti Infective Agents OR Antiinfective Agents OR Agents, Antiinfective OR Anti-Infective Agent OR Agent, Anti-Infective OR Anti Infective Agent OR Microbicides OR Anti-Microbial Agent OR Agent, Anti-Microbial OR Anti Microbial Agent OR Antimicrobial Agents OR Agents, Antimicrobial OR Anti-Microbial Agents OR Agents, Anti-Microbial OR Anti Microbial Agents OR Microbicide OR Antimicrobial Agent OR Agent, Antimicrobial)<br>#6 #4 OR #5<br>#7 randomized controlled trial.pt. OR controlled clinical trial.pt. OR randomized.ti.ab. OR placebo.ti.ab. OR "Clinical Trials as Topic"[Mesh:NoExp] OR trial.ti. OR randomly.ti.ab.<br>#8 #3 AND #6 AND #7
**CENTRAL**	#1 MeSH descriptor: [Intraabdominal Infections] explode all trees and with qualifier(s): [drug therapy - DT]<br>#2 ((Infection, Intraabdominal OR Infections, Intraabdominal OR Intraabdominal Infection OR Intra-Abdominal Infections OR Infection, Intra-Abdominal OR Infections, Intra-Abdominal OR Intra Abdominal Infections OR Intra-Abdominal Infection)):ti,ab,kw<br>#3 MeSH descriptor: [Anti-Infective Agents] explode all trees and with qualifier(s): [therapeutic use - TU]<br>#4 ((Agents, Anti-Infective OR Anti Infective Agents OR Antiinfective Agents OR Agents, Antiinfective OR Anti-Infective Agent OR Agent, Anti-Infective OR Anti Infective Agent OR Microbicides OR Anti-Microbial Agent OR Agent, Anti-Microbial OR Anti Microbial Agent OR Antimicrobial Agents OR Agents, Antimicrobial OR Anti-Microbial Agents OR Agents, Anti-Microbial OR Anti Microbial Agents OR Microbicide OR Antimicrobial Agent OR Agent, Antimicrobial)):ti,ab,kw<br>#5 #1 or #2<br>#6 #3 or #4<br>#7 #5 and #6
Clinicaltrial.gov	intra-abdominal infection

Supplementary table S2 PICOS Table

Domain	Inclusion criteria	Exclusion criteria
Population	Adults aged 18 years of age or older with a cIAI	• Patients aged <18 years<br>• Healthy volunteers<br>• Disease other than disease of our interest<br>• Peritoneal dialysis
Interventions/ Comparators	Antimicrobial regimens that ensured coverage of common pathogens involved in cIAIs	• Aminoglycosides<br>• The same antibiotic class (e.g., meropenem versus imipenem/cilastatin)
Outcomes	• The primary outcome<br>Clinical success rate<br>• Secondary outcomes<br>Microbiological success rate;<br>All-cause mortality;<br>Serious adverse events;<br>Discontinuation due to adverse events	• Outcomes other than listed for inclusion
Study design	RCTs	• Non-RCTs<br>• Single arm trials<br>• Observational studies<br>• Editorials, letters, comments<br>• Case reports, case series<br>• Conference abstract
Restrictions	Articles published in English or Chinese language	• Articles published in non-English or non-Chinese language

Supplementary table S3. General characteristics of studies included in the systematic review

First author	Publication year	NCT	Type of study	Interventions	Sample Size	APACHE II score	Mean Age, year	Sex, female (%)	Duration of therapy, days	Concomitant antibiotic therapy	Fund
Lucasti C [1]	2014	NCT01147640	M, R, DB	Ceftolozane/tazobactam 1.5 g q8h+metronidazole 500 mg q8h	82	Median:7	48.5	37 (45.1)	Mean:5.7	NA	Cubist Pharmaceuticals
				Meropenem 1 g q8h	39	Median:6	46.4	15(38.5)	Mean:6		
Solomkin J [2]	2015	NCT01445665 NCT01445678	M, R, DB	Ceftolozane/tazobactam 1.5 g q8h +metronidazole 500 mg q8h	389	Mean:6.2	50.8	171(44)	4~14	NA	Cubist Pharmaceuticals
				Meropenem 1g q8h	417	Mean:6.0	50.4	169(40.5)			
Solomkin JS [3]	2001	NA	M, R, DB	Clinafloxacin 200 mg q12h	150	Mean:7.9	45.5	54(36.0)	NA	NA	Warner-Lambert
				Imipenem/cilastatin 500 mg q6h	162	Mean:7.8	46.5	59(36.4)			
Solomkin J [4]	2017	NCT01844856	M, R, DB	Eravacycline 1.0mg/kg q12h	220	Mean:6.6	54.9	94(42.7)	Mean:7.6	NA	Tetraphase Pharmaceuticals
				Ertapenem 1.0g q24h	226	Mean:6.8	55.4	94(41.6)	Mean:7.6		
Solomkin JS [5]	2014	NCT01265784	M, R, DB	Eravacycline 1.5mg/kg q24h	56	Mean:8.2	43.6	18(32.1)	Median:6.7	NA	Tetraphase Pharmaceuticals
				Eravacycline 1.0mg/kg q12h	57	Mean:6	42.1	14(24.6)	Median:6.3		
				Ertapenem 1.0g q24h	30	Mean:6.1	41.8	8(26.7)	Median:6.2		
Solomkin JS [6]	2019	NCT018448564	M, R, DB	Eravacycline 1.0mg/kg q12h	195	Mean:6.6	50.3	86 (44.1)	4~14	NA	Tetraphase Pharmaceuticals
				Meropenem 1g q8h	205	Mean:6.4	52.3	100 (48.8)	4~14		
Lucasti C [7]	2016	NCT01506271	M, R, DB	Imipenem/cilastatin+Relebactam 500mg+250mg q6h	83	N (%):80 (96.4) <15	48.3	32(38.6)	NA	NA	Merck
				Imipenem/cilastatin+Relebactam 500mg+150mg q6h	87	N (%): 84 (96.6) <15	49.7	45 (51.7)			
				Placebo+Imipenem/cilastatin 500mg q6h	85	N (%):81 (95.3) <15	48.8	37 (43.5)			
Donahue PE [8]	1998	NA	M, R, DB	Alatrofloxacin 300mg qd switched to oral 200mg alatrofloxacin qd	156	Mean:6.4	43	NA	NA	NA	NA
				Imipenem/cilastatin 500mg q12h/1g q8h switched to oral 500mg amoxicillin/clavulanic q8h	152	Mean:7	45				
Walker AP [9]	1993	NA	M, R, DB	Ampicillin-sulbactam 3g q6h	96	NA	44	17(17.7)	NA	NA	Pfizer
				Cefoxitin 2g q6h	101		46	25(24.8)			
de Groot HG [10]	1993	NA	R, C	Imipenem/cilastatin 500 mg q6h	38	NA	58	18(47.4)	Mean:5	NA	NA
				Aztreonam 1g q8h+clindamycin 600mg q8h	42		64	25(59.5)	Mean:5		

Dela Pena AS [11]	2006	NA	M, R, O	Ertapenem 1g qd	180	Median:2	48	61(33.9)	Median:6	vancomycin or teicoplanin to treat infections caused by resistant gram-positive pathogens	Merck
				Piperacillin/tazobactam 3.375g q6h or 4.5g q8h	190	Median:2	49	70(36.8)	Median:6		
Namias N [12]	2007	NA	M, R, DB	Ertapenem 1g qd	123	Mean:7.2	49.9	90(30.6)	Mean:7	NA	Merck
				Piperacillin/tazobactam 3.375g q6h	108	Mean:6.4	48.7	85(34.7)	Mean:7.6		
Solomkin JS [13]	2003	NA	M, R, DB	Ertapenem 1g qd	323	NA	46.2	130 (40.7)	Mean:7.1	Vancomycin against enterococci or MRSA	Merck
				Piperacillin/tazobactam 3.375g q6h	310		45.4	113 (36.5)	Mean:7.5		
Navarro Jr NS [14]	2005	NA	M, R, O	Ertapenem 1g qd	225	Median:3.0	44	62(27.6)	Median:6	vancomycin or teicoplanin for infections caused by resistant gram-positive pathogens	Merck
				Ceftriaxone 2g/d+metronidazole 30 mg/kg/day	225	Median:3.0	43.9	67(29.8)	Median:6		
Yellin AE [15]	2002a	NA	M, R, DB	Ertapenem 1g qd switched to ciprofloxacin 500mg or 750mg bid+ metronidazole 500mg q8h	59	N (%):44(74.6) <10	37.8	17(29)	Mean:4.6	Vancomycin for enterococcal infections	Merck
				Ceftriaxone 2g/d+metronidazole 500mg q8h switched to ciprofloxacin 500mg or 750mg bid+ metronidazole 500mg q8h	55	N (%):44(76.4) <10	41.1	20(36)	Mean:5.1		
Yellin AE [15]	2002b	NA	M, R, DB	Ertapenem 1.5g qd switched to ciprofloxacin 500mg or 750mg bid+ metronidazole 500mg q8h	51	N (%):34(66.7) <10	45.5	20(39)	Mean:5.3	Vancomycin for enterococcal infections	Merck
				Ceftriaxone 2g/d+metronidazole 500mg q8h switched to ciprofloxacin 500mg or 750mg bid metronidazole 500mg q8h	55	N (%):39(70.9) <10	48.9	20(36)	Mean:6		
Yoshioka K [16]	1991	NA	S, R, C	Ciprofloxacin 200mg q12h+metronidazole 500mg q8h switched to oral ciprofloxacin q12h	40	NA	NA	NA	NA	NA	NA
				Amoxicillin/clavulanic acid 1.2g q8h+metronidazole 500mg q8h switched to oral amoxicillin/clavulanic acid 875mg q8h	40						
Cohn SM [17]	2000	NA	M, R, DB	Ciprofloxacin 400mg q12h+metronidazole 500mg q6h	151	Mean:9.6	47	54(36)	Mean:9	NA	Bayer

				switched to oral ciprofloxacin + metronidazole							
				Piperacillin/tazobactam: 3.375g q6h	131	Mean:9.5	49	49(38)	Mean:10		
Wacha H [18]	2006	NA	M, R, DB	Ciprofloxacin 400mg q12h+metronidazole 500mg q8h switched to oral ciprofloxacin 500mg q12h+ metronidazole 500mg q8h	235	Mean:7.2	52.3	100 (42.6)	Mean:8.5	NA	Bayer
				Ceftriaxone 2g/d+metronidazole 500mg q8h switched to oral metronidazole 500mg q8h	240	Mean:7.6	52.2	95(39.5)	Mean:8.6		
Solomkin JS [19]	1996	NA	M, R, DB	Ciprofloxacin 400mg q12h+metronidazole 500mg q8h	111	Mean:9.2	49.7	66(59.5)	NA	Vancomycin against enterococci or MRSA or antifungal therapy	Miles
				Ciprofloxacin 400mg q12h+metronidazole 500mg q8h switched to oral ciprofloxacin 500mg q12h+ metronidazole 500mg q6h	106	Mean:9.2	52.3	67(63.2)			
				Imipenem/cilastatin 500 mg q6h	113	Mean:10.5	56.1	60(53.1)			
Huizinga WK [20]	1995	NA	M, R, O	Meropenem 1 g q8h	77	N (%):41(53) <5	38	22(28.6)	Mean:6.5	NA	Zeneca
				Cefotaxime 2g q8h+metronidazole 500mg q8h	83	N (%):48(58) <5	35.5	20(24.1)	Mean:6		
Kempf P [21]	1996	NA	M, R, O	Meropenem 1 g q8h	43	N (%):26(60) <10	61.5	21(48.8)	Mean:7.3	NA	Zeneca
				Cefotaxime 2g q8h+metronidazole 500mg q8h	40	N (%):28(70) <10	56.6	16(40)	Mean:6.9		
Chen CW [22]	2013	NCT00952796	S, R, SB	Moxifloxacin 400 mg qd followed oral moxifloxacin 400 mg qd	65	NA	55.5	22(33.8)	Mean:9.6	NA	NA
				Ampicillin/sulbactam 1.5g qid followed oral ampicillin/sulbactam 750mg q12h	65		48.9	29(34.6)	Mean:8.6		
De Waele JJ [23]	2013	NCT00492726	M, R, DB	Moxifloxacin 400 mg qd	352	Mean:6.9	46.7	134 (38.1)	Mean:7	NA	Bayer
				Ertapenem 1g qd	347	Mean:6.8	46.1	116(33.4)	Mean:6.8		
Malangoni MA [24]	2006	NA	M, R, DB	Moxifloxacin 400 mg qd followed oral moxifloxacin 400 mg qd	183	Mean:6.9	47.4	69(37.7)	NA	NA	Bayer
				Piperacillin/tazobactam: 3.375g q6h followed oral amoxicillin/clavulanate 914mg q12h	196	Mean:5.9	45.1	65(49.6)			
Solomkin J [25]	2009	NA	M, R, DB	Moxifloxacin 400 mg qd	180	Mean:3.8	40.3	56(31.1)	Mean:5.8	NA	Bayer
				Ceftriaxone 2g/d+metronidazole	181	Mean:3.8	41.2	55(30.4)	Mean:5.7		

				500mg q12h							
Weiss G [26]	2009	NA	M, R, O	Moxifloxacin 400 mg qd followed oral moxifloxacin 400 mg qd	246	Mean:6.8	48.7	98(40)	Median:5	NA	Bayer
				Ceftriaxone 2g/d+metronidazole 500mg q12h followed oral amoxicillin/clavulanate 625mg q8h	265	Mean:6.6	47.8	108(41)	Median:5		
Ohlin B [27]	1999	NA	M, R, O	Piperacillin/tazobactam 4.5g q8h	140	NA	51	56(40)	Mean:5	NA	NA
				Cefuroxime 1.5g q8h+metronidazole 1.5g q24h	129		54	52(40.3)	Mean:6		
Brismar B [28]	1992	NA	M, R, O	Piperacillin/tazobactam 4.5g q8h	69	NA	52.9	29(42)	Mean:5.5	NA	NA
				Imipenem/cilastatin 1g q8h	65		54	25(38.5)	Mean:5.9		
Erasmo AA [29]	2004	NA	M, R, O	Piperacillin/tazobactam 4.5g q8h	111	NA	42.9	37(33.3)	Mean:5.6	NA	Wyeth Pharmaceuticals
				Imipenem/cilastatin 1g q6h	103		41.3	38(36.9)	Mean:5.5		
Wang HJ [30]	2021	NA	S, R, SB	Meropenem 1g q8h	30	Mean:10.67	63.37	8(26.7)	Mean:8.4	Vancomycin if MRSA or enterococci isolated; Co-infection with fungus in two groups was administered antifungal agents.	NA
				Tigecycline loading dose 100mg followed 50mg q12h	26	Mean:13.12	66.35	5(19.3)	Mean:7.2	cefoperazone-sulbactam for Pseudomonas aeruginosa and Proteus spp;Co-infection with fungus in two groups was administered antifungal agents.	
Qvist N [31]	2012	NCT00230971	M, R, O	Tigecycline loading dose 100mg followed 50mg q12h	232	Mean:6.2	48.55	80(34.5)	Mean:7	NA	Wyeth
				Ceftriaxone 2g/d+metronidazole 1~2g daily	235	Mean:7	46.81	72(30.6)	Mean:6.9		
Towfigh S [32]	2010	NCT00195351	M, R, O	Tigecycline loading dose 100mg followed 50mg q12h	236	N (%):152(64.4) <10	48.17	93(39.4)	NA	NA	Wyeth

				Ceftriaxone 2g/d+metronidazole 1~2g daily	231	N (%):151(65.4) <10	48.79	72(31.2)			
Chen ZJ [33]	2010	NCT00136201	M, R, O	Tigecycline loading dose 100mg followed 50mg q12h	97	Mean:5.1	46.8	32(33)	Median:5	NA	Wyeth
				Imipenem/cilastatin 1g q6h	102	Mean:4.1	41	31(30.4)	Median:6		
Fomin P [34]	2005	NA	M, R, DB	Tigecycline loading dose 100mg followed 50mg q12h	404	Mean:6.44	48.3	165 (40.8)	Mean:7.7	NA	Wyeth
				Imipenem/cilastatin 1g q6h	413	Mean:6.41	49.5	173 (41.9)	Mean:7.8		
Oliva ME [35]	2005	NCT00081744	M, R, DB	Tigecycline loading dose 100mg followed 50mg q12h	247	Mean:5.6	42.9	74(30)	Mean:8.1	NA	Wyeth
				Imipenem/cilastatin 1g q6h	255	Mean:5.5	43.1	89(34.9)	Mean:7.9		
Chen YJ [36]	2018	NCT01721408	M, R, DB	Tigecycline loading dose 100mg followed 50mg q12h	207	Mean:5.2	47.3	75(36.2)	5~14	NA	Pfizer
				Imipenem/cilastatin 1g q6h	205	Mean:5.4	48.7	72(35.1)	5~14		
Barie PS [37]	1997	NA	M, R, DB	Cefepime 2g q12h+metronidazole 500mg or 7.5mg/kg q6h	95	Mean:7.8	49.3	27(28)	Mean:8.8	Vancomycin for enterococcal infections	Bristol-Myers Squibb Company
				Imipenem/cilastatin 500mg q6h	122	Mean:9.3	51.5	40(33)	Mean:9.4		
Garbino J [38]	2007	NA	S, R, DB	Cefepime 2g q12h+metronidazole 500mg q8h	60	Mean:6.2	63	27(44)	Mean:8	NA	Bristol-Myers Squibb Company
				Imipenem/cilastatin 1g q6h	61	Mean:5.5	57	34(56)	Mean:9		
Lucasti C [39]	2013	NCT00752219	M, R, DB	Ceftazidime/avibactam 2.5g q8h+metronidazole 500mg q8h	101	N (%):84(83.2) <10	43	31(30.7)	Median:6	vancomycin, linezolid or daptomycin for suspected or documented methicillin-resistant Staphylococcus aureus (MRSA) and/or enterococcal infections	Pfizer
				Meropenem 1g q8h	102	N (%):85(83.3) <10	42.6	21(20.6)	Median:6.5		
Mazuski JE [40]	2016	NCT01499290 NCT01500239	M, R, DB	Ceftazidime/avibactam 2.5g q8h+metronidazole 500mg q8h	520	N (%):437(84.0) ≤10	49.8	194 (37.3)	Mean:8	Vancomycin, linezolid or daptomycin for suspected or documented methicillin-resistant Staphylococcus aureus (MRSA) and/or	AstraZeneca
				Meropenem 1g q8h	523	N (%):434(83.0) ≤10	50.3	191 (36.5)	Mean:8.3		

										enterococcal infections	
Qin XY [41]	2017	NCT01726023	M, R, DB	Ceftazidime/avibactam 2.5g q8h+metronidazole 500mg q8h	214	N (%):201(93.9) ≤10	48.5	73(34.1)	Mean:6.9	Vancomycin, linezolid or daptomycin for suspected or documented methicillin-resistant Staphylococcus aureus (MRSA) and/or enterococcal infections	AstraZeneca
				Meropenem 1g q8h	217	N (%):201(92.6) ≤10	48.5	64(29.5)	Mean:7.3		
Christou NV [42]	1996	NA	M, R, DB	Cefoxitin 2g q6h	109	Mean:7.7	51.9	NA	NA	NA	Merck Erosst
				Imipenem/cilastatin 1g q6h	104	Mean:8.9	59.7				
Angerås MH [43]	1996	NA	M, R, O	Cefuroxime 3-4g/d+metronidazole 1-1.5g/d	258	N (%):216(83.7) ≤10	54	105 (40.7)	Median:6	NA	NA
				Imipenem/cilastatin 1.5-2g/d	257	N (%):212(82.5) ≤10	56	115 (44.7)	Median:6		
ClinicalTrials.gov 2021 [44]	2021	NCT03830333	M, R, DB	Ceftolozane/tazobactam 1.5 g q8h +metronidazole 500 mg q8h	134	NA	47.5	55(41)	4~14	NA	Merck Sharp
				Meropenem 1 g q8h	134		51.1	49(36.6)	4~14		

Abbreviations: NCT: ClinicalTrials.gov Identifier, M: multicenter, R: randomized, DB: double-blind, S: single-center, O: open label, SB: single-blind, C: controlled, q8h: every 8 hours, q6h: every 6 hours, q12h: every 12 hours, q24h: every 24 hours, qd: once a day, qid: four times a day, bid: twice a day, NA: not available

Supplementary box 1. Antimicrobial regimens eligible for inclusion criteria in the network meta-analysis

Cephalosporin-based regimens

- Ceftriaxone plus Metronidazole (CTX_M)
- Cefotaxime plus Metronidazole (CTX_M)
- Cefuroxime plus Metronidazole (CUX_M)
- Cefepime plus Metronidazole (CEP_M)
- Cefoxitin (COT)

β-lactam/β-lactamase inhibitor-based regimens

- Ceftolozane/Tazobactam plus Metronidazole (TT_M)
- Imipenem/Cilastatin/Relebactam (ICRB)
- Ampicillin/Sulbactam (AS)
- Piperacillin/Tazobactam (PT)
- Amoxicillin/Clavulanic plus Metronidazole (AC_M)
- Ceftazidime/Avibactam plus Metronidazole (CA_M)

Fluoroquinolone -based regimens

- Clinafloxacin (CLI)
- Alatrofloxacin (ALA)
- Moxifloxacin (MOF)
- Ciprofloxacin plus Metronidazole (CIP_M)

Carbapenem regimens

- Meropenem (MEM)
- Imipenem/Cilastatin (IC)
- Ertapenem (ERM)

Other regimens

- Eravacycline (ERA)
- Aztreonam plus Clindamycin (A_C)
- Tigecycline (TGC)

Supplementary table S4. Risk of bias for all included studies

Author	Year	Sequence generation	Allocation sequence concealment	Blinding of participants and personnel	Blinding of outcome assessment	Incomplete outcome data	Selective outcome reporting	Other potential of bias
Lucasti C	2014							
Solomkin J	2015							
Solomkin JS	2001							
Solomkin J	2017							
Solomkin J	2014							
Solomkin J	2019							
Lucasti C	2016							
Donahue PE	1998							
Walker AP	1993							
de Groot HG	1993							
Dela Pena AS	2006							
Namias N	2007							
Solomkin JS	2003							
Navarro Jr NS	2005							
Yellin AE	2002							
Yoshioka K	1991							
Cohn SM	2000							
Solomkin JS	1996							
Huizinga WK	1995							
Kempf P	1996							
Chen CW	2013							
De Waele JJ	2013							
Malangoni MA	2006							
Solomkin J	2009							
Weiss G	2009							
Ohlin B	1999							

Erasmo AA	2004							
Brismar B	1992							
Wang HJ	2021							
Qvist N	2012							
Towfigh S	2010							
Chen ZJ	2010							
Fomin P	2005							
Oliva ME	2005							
Chen YJ	2018							
Garbino J	2007							
Barie PS	1997							
Lucasti C	2013							
Mazuski JE	2016							
Qin XY	2017							
Christou NV	1996							
Angerås MH	1996							
ClinicalTrials.gov	2021							
Wacha H	2006							

Note: Green, low risk; Yellow, unclear risk; Red, high risk

Supplementary table S5. Assessment of Bayesian consistency model fit for all endpoints

Endpoints	DIC*	
	Consistency model	Inconsistency model
Clinical success rates	178.5	183.3
Microbiological success rates	115.1	119.4
All-cause mortality	123.7	126.8
Discontinuation due to adverse events	93.2	99.4

Note: *Once DIC between consistency model and inconsistency model was approximated, it means a good model fit)

Supplementary table S6. Assessment of local inconsistency by node-split method for all endpoints

Outcomes	Comparisons	Direct comparisons results (OR 95%CrI)	Indirect comparisons results (OR 95%CrI)	Network comparisons results (OR 95%CrI)	P values
Clinical success rates	COT vs AS	0.56 (0.24, 1.30)	1.90 (0.45, 7.70)	0.76 (0.37, 1.60)	0.15
	MOF vs AS	1.60 (0.58, 5.00)	0.50 (0.14, 1.70)	1.0 (0.44, 2.30)	0.16
	CTX_M vs CIP_M	0.99 (0.51, 2.00)	0.83 (0.45, 1.50)	0.83 (0.45, 1.50)	0.66
	IC vs CIP_M	0.97 (0.51, 1.80)	0.76 (0.40, 1.40)	0.85 (0.56, 1.30)	0.56
	PT vs CIP_M	0.74 (0.39. 1.40)	1.10 (0.66, 2.10)	0.94 (0.63, 1.50)	0.28
	IC vs COT	1.10 (0.48, 2.40)	3.6 (0.83, 16.00)	1.40 (0.70, 3.00)	0.15
	ERM vs CTX_M	1.40 (0.77, 2.50)	1.10 (0.64. 1.70)	1.20 (0.81, 1.70)	0.49
	MOF vs CTX_M	0.73 (0.45, 1.20)	1.10 (0.65, 2.10)	0.86 (0.61, 1.30)	0.22
	TGC vs CTX_M	0.89 (0.57, 1.40)	0.62 (0.33, 1.10)	0.79 (0.55, 1.10)	0.33
	IC vs CUX_M	0.75 (0.39, 1.40)	1.20 (0.52, 2.50)	0.89 (0.53, 1.50)	0.35
	PT vs CUX_M	1.20 (0.61, 2.30)	0.76 (0.37, 1.70)	0.99 (0.60, 1.70)	0.36
	ERM vs ERA	1.20 (0.66, 2.20)	3.5 (0.58, 24.00)	1.30 (0.76, 2.40)	0.26
	MEM vs ERA	0.88 (0.41, 2.00)	0.31 (0.05, 1.60)	0.75 (0.36, 1.50)	0.28
	MOF vs ERM	0.68 (0.37, 1.20)	0.79 (0.48, 1.30)	0.74 (0.51, 1.10)	0.67
	PT vs ERM	0.95 (0.67, 1.40)	0.78 (0.44, 1.40)	0.91 (0.67, 1.20)	0.54
	PT vs IC	1.40 (0.74, 2.60)	1.00 (0.62, 1.60)	1.10 (0.78, 1.60)	0.43
	TGC vs IC	0.76 (0.54. 1.0)	1.30 (0.66, 2.70)	0.83 (0.62, 1.10)	0.15
	TGC vs MEM	2.50 (0.58, 14.00)	0.86 (0.29, 2.50)	1.20 (0.52, 2.90)	0.24
	PT vs MOF	0.90	1.40	1.20	0.22

		(0.47, 1.70)	(0.91, 2.30)	(0.84, 1.80)	
Microbiological success rates	CTX_M vs CIP_M	0.68<br>(0.21, 2.20)	0.79<br>(0.24. 2.20)	0.75<br>(0.33, 1.50)	0.83
	IC vs CIP_M	1.00<br>(0.30, 3.50)	0.78<br>(0.27, 2.30)	0.88<br>(0.42, 1.80)	0.71
	PT vs CIP_M	0.94<br>(0.31, 2.80)	1.00<br>(0.37, 3.20)	0.98<br>(0.50, 2.00)	0.89
	ERM vs CTX_M	1.40<br>(0.51, 4.00)	0.97<br>(0.35, 2.30)	1.10<br>(0.60, 2.20)	0.56
	MOF vs CTX_M	0.65<br>(0.28. 1.30)	1.40<br>(0.55, 3.80)	0.86<br>(0.47, 1.60)	0.17
	TGC vs CTX_M	1.20<br>(0.40, 3.60)	0.68<br>(0.20, 2.10)	0.92<br>(0.42, 1.90)	0.43
	IC vs CUX_M	0.69<br>(0.24, 1.90)	1.50<br>(0.38, 5.50)	0.89<br>(0.40, 2.00)	0.32
	PT vs CUX_M	1.40<br>(0.46, 4.60)	0.67<br>(0.20, 2.40)	1.00<br>(0.45, 2.40)	0.34
	MOF vs ERM	0.76<br>(0.27, 2.10)	0.78<br>(0.28, 1.90)	0.77<br>(0.40, 1.40)	0.96
	PT vs ERM	1.40<br>(0.60, 3.80)	0.96<br>(0.36, 2.70)	1.20<br>(0.64, 2.40)	0.53
	PT vs IC	1.20<br>(0.47, 3.40)	1.00<br>(0.41, 2.60)	1.10<br>(0.60, 2.10)	0.78
	TGC vs IC	0.73<br>(0.35, 1.20)	1.30<br>(0.28, 5.40)	0.79<br>(0.42, 1.20)	0.42
	PT vs MOF	0.96<br>(0.37, 2.00)	2.20<br>(1.00, 6.20)	1.50<br>(0.84, 3.20)	0.14
All-cause mortality	CTX_M vs CIP_M	0.97<br>(0.37, 2.50)	0.94<br>(0.31, 2.70)	0.96<br>(0.48, 1.80)	0.97
	IC vs CIP_M	1.10<br>(0.39, 3.00)	1.20<br>(0.35, 4.00)	1.10<br>(0.53, 2.40)	0.91
	PT vs CIP_M	0.79<br>(0.28, 2.20)	0.81<br>(0.26, 2.70)	0.80<br>(0.38, 1.70)	0.96
	ERM vs CTX_M	2.70<br>(0.51, 26.00)	0.60<br>(0.24, 1.40)	0.83<br>(0.39, 1.80)	0.052
	MOF vs CTX_M	0.80<br>(0.29, 2.10)	1.40<br>(0.44, 4.70)	1.00<br>(0.49, 2.10)	0.42
	TGC vs CTX_M	1.50<br>(0.59, 4.20)	2.1<br>(0.56, 8.40)	1.70<br>(0.82, 3.60)	0.71
	IC vs CUX_M	1.60<br>(0.63, 4.40)	0.83<br>(0.10, 6.20)	1.50<br>(0.61, 3.20)	0.54
	PT vs CUX_M	0.69<br>(0.11, 3.60)	1.30<br>(0.34, 5.70)	1.00<br>(0.36, 2.90)	0.55
	MOF vs ERM	1.80<br>(0.75, 4.50)	0.71<br>(0.26. 1.90)	1.20<br>(0.62, 2.30)	0.16
	PT vs ERM	0.96<br>(0.54, 1.80)	1.10<br>(0.33, 3.70)	1.00<br>(0.60, 1.70)	0.80
	TGC vs IC	1.50<br>(0.76, 3.00)	1.10<br>(0.23, 5.00)	1.40<br>(0.77, 2.60)	0.71
	PT vs MOF	1.20<br>(0.33, 4.30)	0.69<br>(0.30, 1.70)	0.82<br>(0.42, 1.70)	0.49
Discontinuation due to adverse events	CTX_M vs CIP_M	1.50<br>(0.48, 5.20)	1.30<br>(0.36, 5.20)	1.40<br>(0.61, 3.60)	0.89
	PT vs CIP_M	1.20<br>(0.50, 3.00)	1.40<br>(0.27, 6.70)	1.20<br>(0.59, 2.70)	0.90
	ERM vs	0.98	0.61	0.72	0.65

	CTX_M	(0.15, 5.70)	(0.20, 1.90)	(0.29, 1.70)	
	MOF vs CTX_M	1.40 (0.27, 7.80)	1.00 (0.32, 3.20)	1.20 (0.45, 2.70)	0.73
	TGC vs CTX_M	1.60 (0.85, 2.90)	3.40 (0.45, 33.00)	1.60 (0.93, 3.00)	0.49
	IC vs CUX_M	1.30 (0.60, 3.10)	1.50 (0.81, 2.10)	1.50 (0.67, 3.30)	0.37

Supplementary table S7. Network meta-analyses and pairwise meta-analyses for all endpoints

		Network meta-analyses	Pairwise meta-analyses		
Clinical success rates					
Contrast	Group	OR 95%CrI	N	OR 95%CI	I^2 (%)
IC *vs*	TGC	1.20 (0.91, 1.61)	4	1.33 (0.91, 2.08)	43.0
	CEP_M	0.61 (0.34, 1.10)	2	0.60 (0.30, 1.20)	0
	CIP_M	0.85 (0.56, 1.30)	1	0.97 (0.44, 2.10)	-
	CLI	0.78 (0.42, 1.50)	1	0.79 (0.35, 1.80)	-
	ALA	1.10 (0.52, 2.20)	1	1.10 (0.43, 2.60)	-
	A_C	1.40 (0.50, 4.20)	1	1.40 (0.44, 4.40)	-
	COT	1.40 (0.70, 2.90)	1	1.10 (0.41, 2.80)	-
	CUX_M	0.89 (0.53, 1.50)	1	0.74 (0.33, 1.70)	-
	ICRB	0.77 (0.33, 1.89)	1	0.77 (0.29, 2.13)	-
	PT	0.91 (0.63,1.28)	2	0.71 (0.33, 1.43)	88.8
MEM *vs*	TGC	0.83 (0.34, 1.96)	1	0.40 (0.07, 2.00)	51.2
	CA_M	1.20 (0.83, 1.90)	3	1.20 (0.75, 2.10)	0.0
	COX_M	1.30 (0.43, 3.60)	2	1.20 (0.39, 3.80)	84.7
	ERA	0.75 (0.36, 1.50)	1	0.89 (0.35, 2.30)	-
	TT_M	1.32 (0.83, 2.08)	3	1.32 (0.77, 2.38)	0.0
PT *vs*	MOF	1.20 (0.84, 1.80)	1	0.90 (0.39, 2.10)	-
	CIP_M	0.94 (0.63, 1.50)	1	0.74 (0.32, 1.70)	-
	CUX_M	0.98 (0.60, 1.70)	1	1.20 (0.52, 2.80)	-
	ERM	0.91 (0.67, 1.20)	3	0.96 (0.61, 1.50)	0.0
ERM *vs*	ERA	1.30 (0.77, 2.40)	2	1.20 (0.60, 2.50)	0.0
	MOF	1.37 (0.91, 1.96)	1	1.47 (0.67, 3.23)	-
	CTX_M	1.20 (0.82, 1.70)	3	1.40 (0.73, 2.70)	0.0
MOF *vs*	AS	0.99 (0.45, 2.30)	1	1.60 (0.50, 5.40)	-
	CTX_M	0.86 (0.61, 1.30)	2	0.72 (0.39, 1.30)	0.0
CIP_M *vs*	AC_M	5.50 (0.64, 2.62)	1	5.30 (0.55, 1.72)	-
COT *vs*	AS	0.77 (0.37, 1.60)	1	0.56 (0.20, 1.50)	-
CTX_M *vs*	CIP_M	0.90 (0.58,1.40)	1	1.00 (0.44, 2.20)	-
TGC *vs*	CTX_M	0.79 (0.55, 1.10)	2	0.90 (0.52, 1.60)	52.6
Microbiological success rates					
Contrast	Group	OR 95%CrI	N	OR 95%CI	I^2 (%)
IC *vs*	CEP_M	0.69 (0.33, 1.40)	2	0.68 (0.21, 2.20)	0.0
	CIP_M	0.87 (0.42, 1.80)	1	1.00 (0.18, 5.70)	-
	CUX_M	0.90 (0.41, 2.00)	1	0.69 (0.13, 3.60)	-
	ICRB	0.83 (1.61, 1.23)	1	0.83 (0.10,7.69)	-
	PT	0.91 (0.48, 1.67)	2	0.77 (0.20, 2.70)	84.6
	TGC	1.27 (0.83, 2.33)	4	1.54 (0.71, 4.35)	67.7
CTX_M *vs*	CIP_M	0.74 (0.34, 1.50)	1	0.67 (0.12, 3.70)	-
	ERM	0.91 (0.48, 1.67)	3	0.71 (0.2, 2.44)	0.0
	MOF	1.15 (0.66, 2.13)	2	1.64 (0.53,5.88)	62.5

	TGC	1.08 (0.53, 2.38)	1	0.83 (0.16, 4.35)	-
MOF *vs*	PT	0.65 (0.31, 1.18)	1	1.04 (0.21, 5.26)	-
	ERM	0.77 (0.41, 1.40)	1	0.76 (0.16, 3.70)	-
PT *vs*	CIP_M	0.97 (0.50, 2.00)	1	0.93 (0.18, 4.70)	-
	CUX_M	1.00 (0.45, 2.40)	1	1.40 (0.26, 7.90)	-
	ERM	1.20 (0.65, 2.40)	2	1.50 (0.46, 6.30)	61.9
ERM *vs*	ERA	1.20 (0.26, 7.00)	1	1.20 (0.16, 10.00)	-
TT_M *vs*	MEM	-	2	0.89 (0.26, 3.03)	0.0
CA_M *vs*	MEM	-	2	0.77 (0.54, 1.10)	0.0
COX_M *vs*	MEM	-	2	0.82 (0.21, 3.19)	0.0
All-cause mortality					
Contrast	Group	OR 95%CrI	N	OR 95%CI	I^2 (%)
MOF *vs*	CTX_M	1.00 (0.49, 2.20)	2	0.80 (0.26, 2.40)	0.0
	ERM	1.20 (0.61, 2.40)	1	1.80 (0.65, 5.30)	-
MEM *vs*	CA_M	0.60 (0.25, 1.40)	3	0.59 (0.23, 1.50)	0.0
	COX_M	0.72 (0.24, 2.00)	2	0.71 (0.22, 2.10)	0.0
	TGC	0.83 (0.12, 5.88)	1	0.83 (0.11, 6.25)	-
	TT_M	0.67 (0.24, 1.69)	3	0.67 (0.22, 1.85)	43 4
IC *vs*	CEP_M	3.30 (0.96, 13.0)	2	3.3 (0.91, 14.00)	0.0
	CIP_M	1.20 (0.54, 2.50)	1	1.10 (0.36, 3.40)	-
	CLI	0.58 (0.14, 2.10)	1	0.57 (0.14, 2.20)	-
	COT	0.98 (0.09, 11.00)	1	1.00 (0.10, 12.00)	-
	CUX_M	1.50 (0.60, 3.30)	1	1.60 (0.56, 4.80)	-
	TGC	0.71 (0.37, 1.30)	4	0.67 (0.31, 1.37)	0.0
CTX_M *vs*	CIP_M	0.97 (0.48, 1.90)	1	0.98 (0.35, 2.80)	-
	ERM	1.22 (0.53, 2.70)	2	0.83 (0.04, 2.08)	0.0
	TGC	0.59 (0.27, 1.22)	2	0.67 (0.24, 1.72)	0.0
PT *vs*	CIP_M	0.81 (0.38, 1.70)	1	0.81 (0.24, 2.50)	-
	CUX_M	1.00 (0.34, 2.90)	1	0.69 (0.09, 3.90)	-
	ERM	1.00 (0.59, 1.70)	3	0.98 (0.51, 2.00)	47.6
	MOF	0.82 (0.41, 1.70)	1	1.20 (0.30, 4.70)	-
ERM *vs*	ERA	1.30 (0.37, 4.70)	1	1.30 (0.31, 5.00)	-
Serious adverse events					
Contrast	Group	OR 95%CrI	N	OR 95%CI	I^2 (%)
TT_M *vs*	MEM	-	3	1.25 (0.74, 2.12)	16.0
TGC *vs*	CTX_M	-	2	1.01 (0.72, 1.40)	0.0
	IC	-	4	**1.57 (1.07, 2.32)** *	46.0
PT *vs*	IC	-	1	0.57 (0.13, 2.44)	-
MOF *vs*	ERM	-	1	1.23 (0.82, 1.85)	-
	PT	-	1	0.94 (0.64, 1.38)	-
	CTX_M	-	1	**4.24 (1.18, 15.28)** *	-
ERM	CTX_M	-	1	**1.93 (1.06, 3.50)** *	-
Discontinuation due to adverse events					
Contrast	Group	OR 95%CrI	N	OR 95%CI	I^2 (%)
MEM *vs*	CA_M	**0.48 (0.23, 0.97)** *	3	**0.48 (0.22, 0.99)** *	0.0
	ERA	0.99 (0.20, 4.80)	1	0.97 (0.20, 5.00)	-
	TT_M	1.18 (0.38, 4.17)	3	1.18 (0.37, 4.00)	0.0
CTX_M *vs*	CIP_M	1.40 (0.56, 3.50)	1	1.50 (0.45, 5.30)	-
	ERM	1.37 (0.56, 3.70)	1	1.01 (0.17, 6.25)	-
	MOF	0.83 (0.33, 2.17)	1	0.71 (0.12, 4.35)	-
PT *vs*	CIP_M	1.30 (0.58, 2.70)	1	1.20 (0.49, 3.10)	-

	ERM	1.20 (0.68, 2.20)	3	1.20 (0.59, 2.60)	0.0
	IC	0.70 (0.25, 1.90)	1	0.18 (0.0072,1.60)	-
	MOF	0.76 (0.42,1.30)	1	0.81 (0.38,1.80)	-
IC *vs*	CLI	1.60 (0.36, 8.60)	1	1.60 (0.36, 9.80)	-
	CUX_M	1.50 (0.67, 3.30)	1	1.30 (0.56, 3.30)	-
	ICRB	1.00 (0.18, 4.35)	1	1.00 (0.15, 4.17)	-
	TGC	0.77 (0.50,1.18)	4	0.77 (0.45, 1.22)	0.0
ERM *vs*	ERA	2.50 (0.78, 9.30)	1	2.60 (0.77, 9.90)	-
	MOF	0.63 (0.31, 1.22)	1	0.56 (0.16, 1.59)	-

Note: Bold results with mark of * indicate statistical significance

Supplementary table S8. Ranking of interventions for all endpoints by Surface Under the Cumulative Ranking (SUCRA) Curve

Interventions	Clinical success rates		Microbiological success rates		All-cause mortality		Discontinuation due to adverse event	
	SUCRA	Rank	SUCRA	Rank	SUCRA	Rank	SUCRA	Rank
CEP_M	91.2	1	82.8	1	86.0	1	-	-
ERM	76.6	3	51.8	6	65.9	4	55.9	5
COX_M	12.0	21	-	-	30.1	13	47.9	8
CLI	78.1	2	-	-	24.2	15	50.8	7
ICRB	74.8	4	59.6	3	-	-	33.9	12
CIP_M	72.6	5	63.7	2	50.2	9	57.3	4
CUX_M	67.3	6	58.6	4	64.9	6	47.7	9
PT	63.7	7	54.7	5	65.5	5	44.5	10
ERA	50.8	10	45.4	8	77.6	2	84.0	1
MEM	36.0	17	-	-	43.2	11	80.7	3
AS	50.6	12	-	-	74.6	3	-	-
IC	56.7	9	47.4	7	42.1	12	28.6	13
MOF	42.0	13	19.4	11	50.6	8	28.5	14
CTX_M	62 4	8	34.2	9	51.5	7	39.5	11
ALA	52.5	10	-	-	-	-	-	-
CA_M	22.1	18	-	-	22.1	17	54.5	6
A_C	36.4	16	-	-	-	-	-	-
TT_M	19.8	19	-	-	29.3	14	83.3	2
TGC	36.6	15	32.2	10	24.7	16	12.9	15
COT	30.2	16	-	-	47.6	10	-	-
AC_M	17.5	20	-	-	-	-	-	-

Supplementary table S9. Network regression for publication year

Outcomes		95%CrI #
Clinical success rates	beta [1]	-32.1~15.3
	beta [2]	-26.4~8.2
	beta [3]	-23.7~ 30.9
	beta [4]	-3.3~ 2.3
	beta [5]	-3.2~ 6.2
	beta [6]	-1.2~ 2.8
	beta [7]	-2.2~1.2
	beta [8]	-22.1~ 16.7
	beta [9]	-7.4~ 25.3
	beta [10]	-80.9~4.7
	beta [11]	-2.02~ 2.07
	beta [12]	-5.5 ~3.1
	beta [13]	-5.8~4.1
	beta [14]	-3.0~0.5
	beta [15]	-13.0~12.3
	beta [16]	-4.3~4.2
	beta [17]	-4.8~ 0.9
	beta [18]	-2.9~0.19
	beta [19]	-1.4~0.6
	beta [20]	-4.9~3.7
Microbiological success rates	beta [1]	-0.8~2.7
	beta [2]	-1.8~2.3
	beta [3]	-1.1~3.7
	beta [4]	-4.6~3.0
	beta [5]	-10.5~6.7
	beta [6]	-2.1~2.2
	beta [7]	-0.05~2.1
	beta [8]	-8.6~7.03
	beta [9]	-3.1~1.6
	beta [10]	-2.6~0.8
All-cause mortality	beta [1]	-11.4~13.1
	beta [2]	-4.7~4.9
	beta [3]	-2.4~4.6
	beta [4]	-2.6~1.9
	beta [5]	-9.3~9.9
	beta [6]	-8.2~5.7
	beta [7]	-5.5~11.5
	beta [8]	-2.0~4.3
	beta [9]	-4.1~6.3
	beta [10]	-12.7~3.0
	beta [11]	-3.9~0.8
	beta [12]	-1.6~1.2
	beta [13]	-3.4~4.2
	beta [14]	-2.8~3.3
	beta [15]	-1.3~3.9
	beta [16]	-59.2~2.5
Discontinuation due to adverse events	beta [1]	-3.4~4.4
	beta [2]	-3.7~2.8
	beta [3]	-8.8~26.6
	beta [4]	-27.6~6.3
	beta [5]	-1.9~4.6

	beta [6]	-9.2~3.6
	beta [7]	-4.5~7.2
	beta [8]	-2.2~2.5
	beta [9]	-1.1~0.9
	beta [10]	-23.3~10.1
	beta [11]	-3.4~2.6
	beta [12]	-2.2~3.4
	beta [13]	-3.4~2.1
	beta [14]	-3.4~4.1

Note: #, When 95% CrI contains zero, it means publication year had no any potential moderating association with treatments

Supplementary figure S1A. The trace plot and the Brooks-Gelman-Rubin plot of clinical success rates

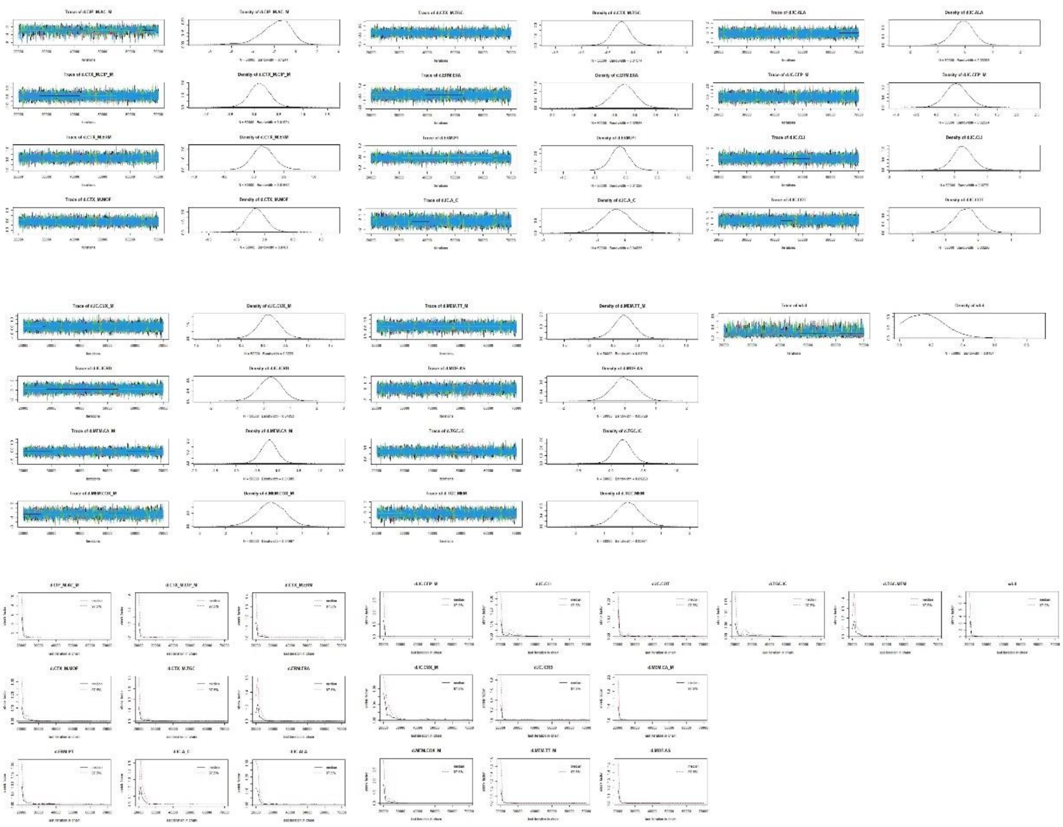

Supplementary figure S1B. The trace plot and the Brooks-Gelman-Rubin plot of microbiological success rates

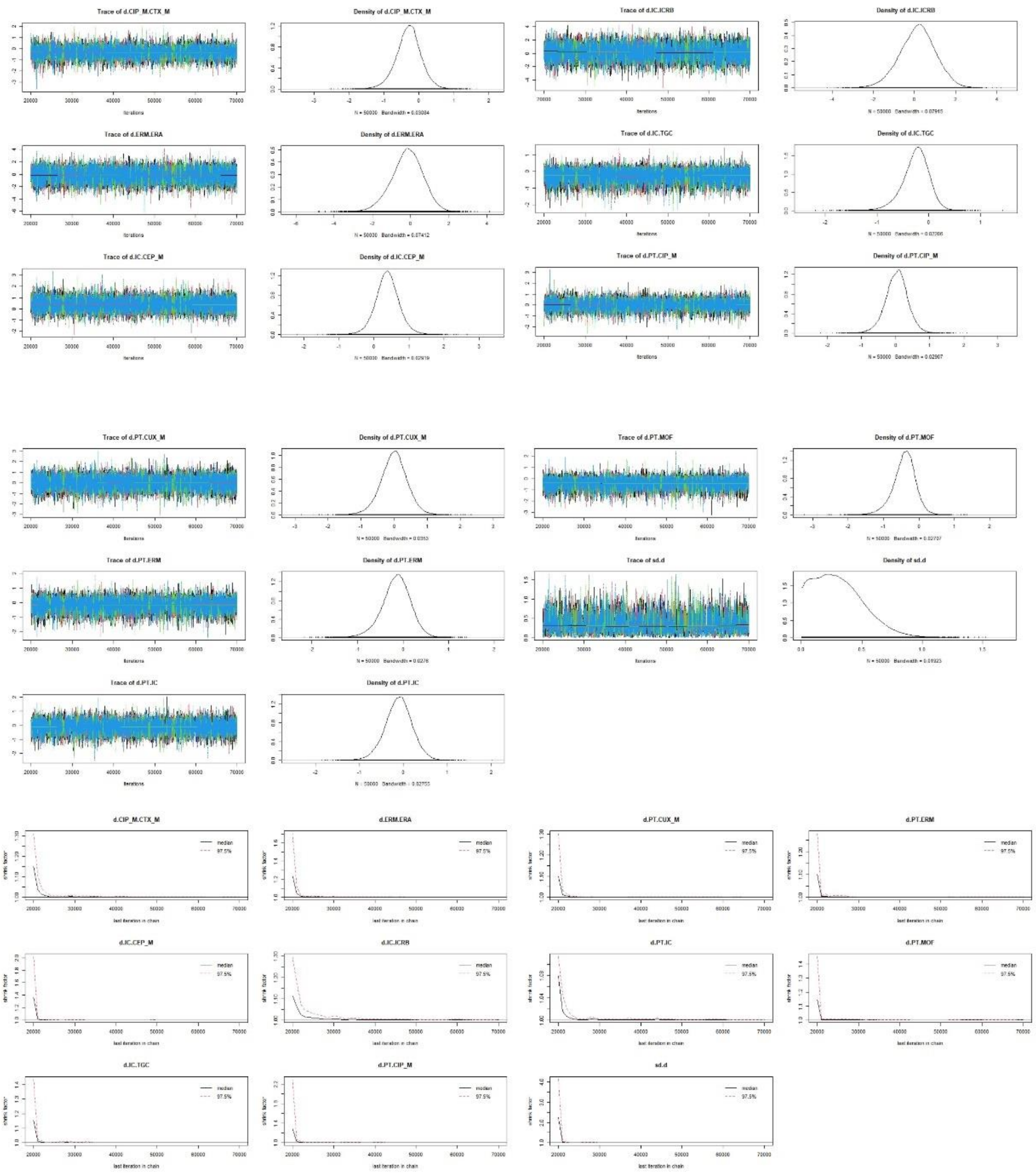

Supplementary figure S1C. The trace plot and the Brooks-Gelman-Rubin plot of all-cause mortality

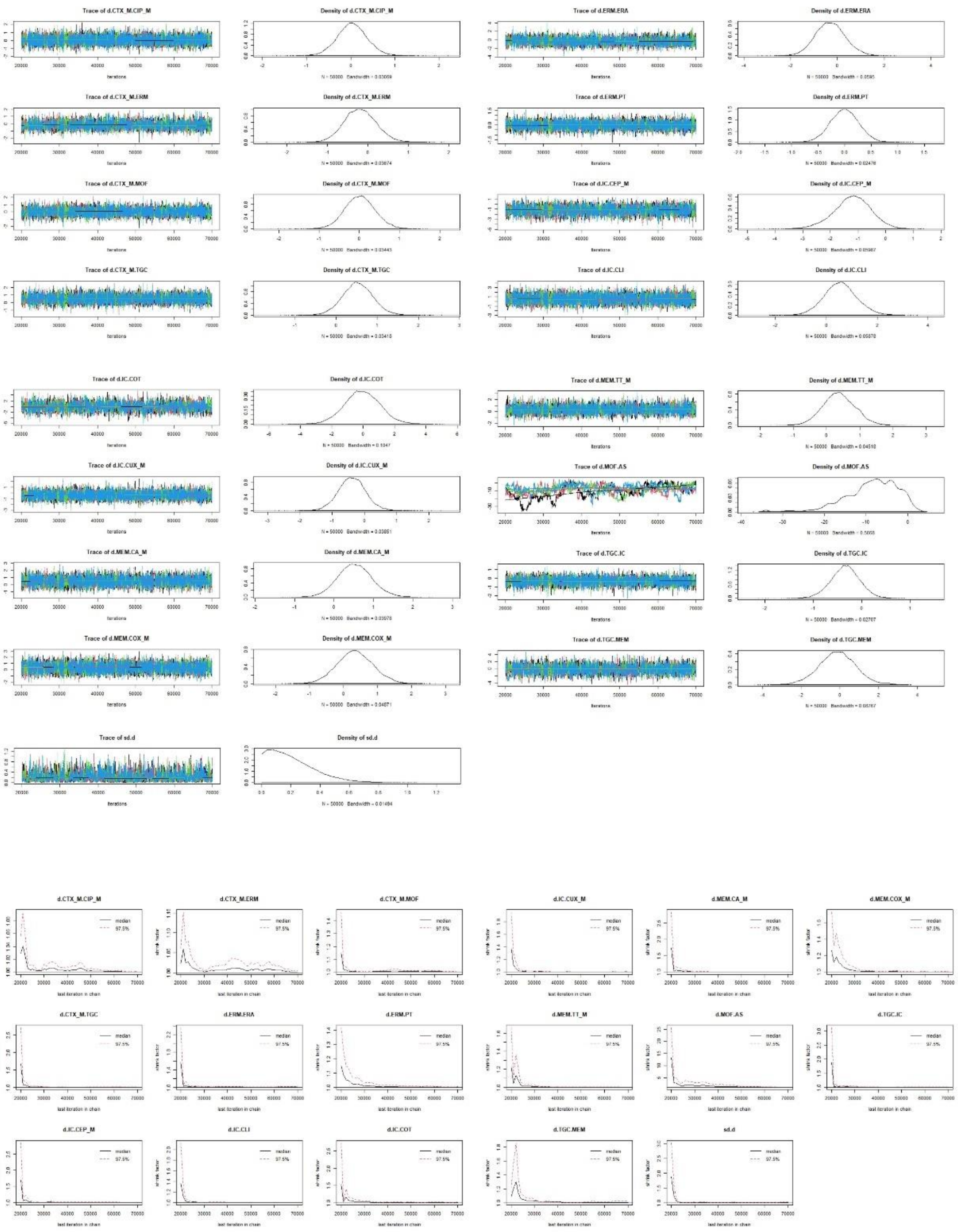

Supplementary figure S1D. The trace plot and the Brooks-Gelman-Rubin plot of discontinuation due to adverse events

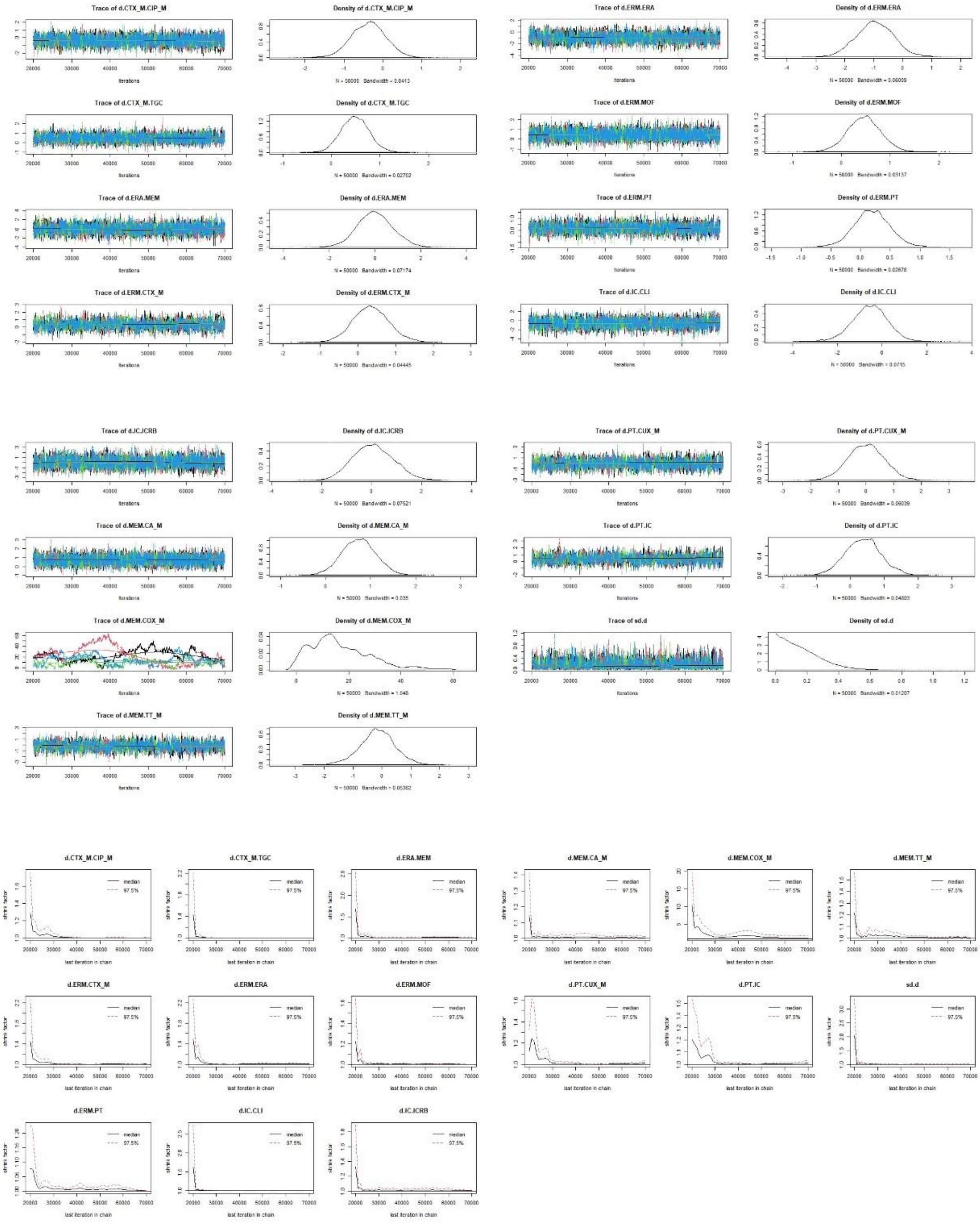

Supplementary league table S1 Network meta-analysis results for clinical success rates based on data from patients with APACHE II score >/= 10

MEM								
5.4(0.59,70.0)	COX_M							
0.99(0.073,14.0)	0.18(0.0048,5.5)	MOF						
1.6(0.43,5.8)	0.29(0.017,3.8)	1.6(0.084,29.0)	CA_M					
1.1(0.038,28.0)	0.19(0.003,9.9)	1.1(0.14,7.5)	0.66(0.019,23.0)	ERM				
1.3(0.23,8.1)	0.24(0.011,4.3)	1.3(0.057,33.0)	0.84(0.093,7.8)	1.3(0.031,57.0)	TT_M			
1.4(0.067,31.0)	0.25(0.005,12.0)	1.4(0.29,7.1)	0.89(0.033,26.0)	1.4(0.42,4.7)	1.1(0.03,37.0)	PT		
1.4(0.16,12.0)	0.25(0.009,5.6)	1.4(0.32,6.3)	0.88(0.072,11.0)	1.3(0.11,17.0)	1.1(0.062,17.0)	0.99(0.11,8.7)	CTX_M	
1.5(0.25,9.6)	0.28(0.013,4.9)	1.5(0.24,10.0)	0.96(0.10,9.2)	1.5(0.098,24.0)	1.1(0.0088,14.0)	1.1(0.091,13.0)	1.1(0.35,3.4)	TGC

Note: Intervention effects, measured as odds ratios of interventions for clinical success rate. Odds ratios higher than 1 favor the column-defining intervention for the network meta-analysis results.

Supplementary league table S1A. The result of sensitivity analysis excluding study evaluating trovafloxacin for clinical success rates

A_C																			
4.03 (0.29, 127.05)	AC_M																		
0.81 (0.21, 3.02)	0.2 (0.01, 2.43)	AS																	
1.31 (0.31, 5.56)	0.33 (0.01, 4.16)	1.62 (0.46, 5.91)	CA_M																
0.45 (0.13, 1.43)	0.11 (0, 1.23)	0.55 (0.2, 1.5)	0.34 (0.11, 1.02)	CEP_M															
0.63 (0.19, 1.87)	0.16 (0.01, 1.53)	0.77 (0.31, 1.91)	0.47 (0.17, 1.27)	1.4 (0.69, 2.83)	CIP_M														
0.57 (0.16, 1.88)	0.15 (0.01, 1.61)	0.7 (0.25, 1.98)	0.43 (0.13, 1.36)	1.27 (0.56, 3.02)	0.9 (0.44, 1.95)	CLI													
1.07 (0.28, 3.75)	0.27 (0.01, 3.07)	1.3 (0.63, 2.7)	0.8 (0.24, 2.6)	2.35 (0.96, 6.03)	1.69 (0.77, 3.89)	1.86 (0.73, 4.82)	COT												
1.37 (0.23, 7.79)	0.34 (0.01, 5.18)	1.69 (0.34, 8.24)	1.04 (0.33, 3.19)	3.05 (0.69, 13.82)	2.2 (0.53, 9.21)	2.43 (0.52, 11.12)	1.3 (0.27, 6)	COX_M											
0.7 (0.22, 2.05)	0.18 (0.01, 1.8)	0.85 (0.36, 2.05)	0.53 (0.2, 1.37)	1.55 (0.8, 3.11)	1.11 (0.74, 1.72)	1.23 (0.59, 2.5)	0.66 (0.3, 1.41)	0.51 (0.13, 2)	CTX_M										
0.66 (0.19, 2.03)	0.17 (0.01, 1.76)	0.8 (0.31, 2.11)	0.5 (0.17, 1.4)	1.46 (0.69, 3.17)	1.04 (0.58, 1.94)	1.15 (0.51, 2.53)	0.62 (0.26, 1.46)	0.48 (0.11, 2.01)	0.94 (0.53, 1.65)	CUX_M									
0.79 (0.22, 2.69)	0.2 (0.01, 2.16)	0.97 (0.35, 2.74)	0.6 (0.26, 1.34)	1.74 (0.75, 4.37)	1.26 (0.62, 2.62)	1.39 (0.56, 3.47)	0.74 (0.29, 1.94)	0.57 (0.16, 2.07)	1.13 (0.59, 2.19)	1.2 (0.56, 2.68)	ERA								
0.6 (0.18, 1.78)	0.15 (0.01, 1.54)	0.74 (0.3, 1.76)	0.45 (0.17, 1.14)	1.33 (0.66, 2.72)	0.95 (0.6, 1.52)	1.06 (0.49, 2.18)	0.57 (0.25, 1.22)	0.43 (0.11, 1.68)	0.86 (0.59, 1.23)	0.91 (0.52, 1.6)	0.76 (0.43, 1.32)	ERM							
0.74 (0.24, 2.05)	0.19 (0.01, 1.9)	0.9 (0.39, 2.13)	0.56 (0.21, 1.45)	1.64 (0.94, 2.94)	1.17 (0.78, 1.79)	1.3 (0.69, 2.39)	0.69 (0.34, 1.41)	0.53 (0.13, 2.14)	1.06 (0.73, 1.54)	1.12 (0.68, 1.86)	0.94 (0.47, 1.81)	1.23 (0.82, 1.88)	IC						
0.58 (0.14, 2.27)	0.15 (0, 1.71)	0.71 (0.21, 2.42)	0.43 (0.12, 1.6)	1.28 (0.47, 3.68)	0.92 (0.36, 2.42)	1.02 (0.35, 2.89)	0.54 (0.18, 1.65)	0.42 (0.08, 2.18)	0.83 (0.33, 2.14)	0.88 (0.33, 2.39)	0.73 (0.24, 2.18)	0.96 (0.38, 2.53)	0.78 (0.34, 1.85)	ICRB					
1.05 (0.27, 4.09)	0.27 (0.01, 3.2)	1.31 (0.4, 4.32)	0.8 (0.53, 1.2)	2.34 (0.85, 6.9)	1.69 (0.69, 4.33)	1.87 (0.64, 5.49)	1 (0.33, 3.05)	0.77 (0.26, 2.25)	1.52 (0.64, 3.69)	1.61 (0.63, 4.39)	1.35 (0.67, 2.74)	1.77 (0.78, 4.14)	1.44 (0.61, 3.52)	1.84 (0.54, 6.44)	MEM				
0.82 (0.25, 2.43)	0.21 (0.01, 2.09)	1 (0.43, 2.29)	0.61 (0.22, 1.61)	1.81 (0.88, 3.72)	1.29 (0.79, 2.11)	1.43 (0.65, 3)	0.77 (0.34, 1.64)	0.59 (0.15, 2.36)	1.17 (0.8, 1.64)	1.24 (0.68, 2.23)	1.03 (0.52, 1.97)	1.36 (0.93, 1.97)	1.1 (0.7, 1.69)	1.41 (0.53, 3.62)	0.76 (0.31, 1.83)	MOF			
0.66 (0.2, 1.94)	0.17 (0.01, 1.69)	0.81 (0.34, 1.94)	0.5 (0.19, 1.29)	1.48 (0.75, 2.9)	1.06 (0.69, 1.6)	1.17 (0.55, 2.33)	0.62 (0.28, 1.34)	0.48 (0.12, 1.89)	0.95 (0.64, 1.36)	1.01 (0.6, 1.66)	0.84 (0.44, 1.55)	1.1 (0.81, 1.48)	0.9 (0.61, 1.28)	1.15 (0.44, 2.85)	0.62 (0.26, 1.46)	0.82 (0.55, 1.19)	PT		
0.89 (0.28, 2.55)	0.23 (0.01, 2.31)	1.08 (0.46, 2.61)	0.67 (0.25, 1.73)	**1.97 (1.06, 3.79)**	1.41 (0.91, 2.25)	1.56 (0.77, 3.07)	0.83 (0.39, 1.77)	0.64 (0.16, 2.56)	1.27 (0.89, 1.81)	1.35 (0.78, 2.37)	1.13 (0.56, 2.18)	1.47 (0.97, 2.3)	1.2 (0.91, 1.6)	1.54 (0.62, 3.74)	0.84 (0.35, 1.96)	1.09 (0.71, 1.73)	1.33 (0.9, 2.06)	TGC	
1.38 (0.33, 5.82)	0.35 (0.01, 4.38)	1.72 (0.48, 6.07)	1.05 (0.58, 1.93)	**3.1 (1.03, 10.03)**	2.23 (0.83, 6.34)	2.46 (0.79, 7.91)	1.32 (0.4, 4.45)	1.01 (0.32, 3.32)	2 (0.77, 5.41)	2.12 (0.76, 6.4)	1.77 (0.78, 4.12)	2.33 (0.93, 6.13)	1.9 (0.73, 5.16)	2.41 (0.66, 9.1)	1.31 (0.86, 2.07)	1.72 (0.65, 4.75)	2.11 (0.82, 5.72)	1.58 (0.61, 4.23)	TT_M

Note: Data are OR (95% CrI) of the row treatment relative to the column treatment in terms of clinical success rates. Bold values indicate comparisons that are statistically significant. ORs above 1 indicate higher rates of clinical success rates.

Supplementary league table S1B. The result of sensitivity analysis excluding studies with high-risk bias of blinding for clinical success rates

ALA																	
1.01 (0.35, 2.86)	AS																
1.22 (0.34, 4.37)	1.19 (0.32, 4.39)	CA_M															
0.57 (0.24, 1.36)	0.56 (0.21, 1.53)	0.47 (0.14, 1.56)	CEP_M														
0.79 (0.36, 1.72)	0.78 (0.33, 1.81)	0.66 (0.21, 1.92)	1.37 (0.72, 2.64)	CIP_M													
0.73 (0.3, 1.77)	0.71 (0.27, 1.96)	0.6 (0.18, 2.03)	1.26 (0.59, 2.78)	0.92 (0.46, 1.85)	CLI												
1.33 (0.5, 3.53)	1.32 (0.66, 2.66)	1.11 (0.31, 3.91)	2.32 (0.98, 5.62)	1.68 (0.79, 3.67)	1.82 (0.74, 4.72)	COT											
0.88 (0.4, 1.9)	0.87 (0.39, 1.94)	0.73 (0.25, 2.09)	1.53 (0.81, 2.9)	1.11 (0.76, 1.65)	1.2 (0.61, 2.45)	0.66 (0.31, 1.38)	CTX_M										
0.83 (0.36, 1.85)	0.81 (0.33, 1.97)	0.68 (0.22, 2.09)	1.43 (0.72, 2.94)	1.04 (0.61, 1.8)	1.13 (0.54, 2.38)	0.62 (0.27, 1.38)	0.94 (0.56, 1.57)	CUX_M									
0.88 (0.33, 2.28)	0.87 (0.31, 2.3)	0.73 (0.31, 1.65)	1.53 (0.65, 3.64)	1.12 (0.54, 2.22)	1.21 (0.48, 2.97)	0.66 (0.25, 1.66)	1 (0.51, 1.92)	1.07 (0.49, 2.27)	ERA								
0.74 (0.33, 1.61)	0.73 (0.32, 1.66)	0.61 (0.22, 1.63)	1.28 (0.66, 2.49)	0.93 (0.6, 1.42)	1.01 (0.49, 2.04)	0.55 (0.26, 1.18)	0.84 (0.59, 1.18)	0.9 (0.53, 1.5)	0.84 (0.48, 1.47)	ERM							
0.93 (0.47, 1.85)	0.92 (0.42, 2.05)	0.78 (0.26, 2.26)	1.63 (0.97, 2.81)	1.18 (0.82, 1.75)	1.28 (0.72, 2.3)	0.7 (0.35, 1.39)	1.07 (0.75, 1.51)	1.13 (0.72, 1.81)	1.06 (0.55, 2.15)	1.26 (0.86, 1.91)	IC						
0.74 (0.25, 2.15)	0.72 (0.23, 2.23)	0.61 (0.16, 2.38)	1.27 (0.48, 3.45)	0.92 (0.38, 2.35)	1 (0.37, 2.78)	0.55 (0.19, 1.6)	0.83 (0.34, 2.09)	0.89 (0.35, 2.35)	0.83 (0.29, 2.51)	0.99 (0.4, 2.54)	0.78 (0.35, 1.82)	ICRB					
0.98 (0.29, 3.3)	0.96 (0.27, 3.33)	0.81 (0.55, 1.16)	1.71 (0.55, 5.34)	1.23 (0.45, 3.48)	1.34 (0.42, 4.31)	0.73 (0.22, 2.45)	1.1 (0.41, 3.02)	1.18 (0.41, 3.45)	1.11 (0.53, 2.36)	1.31 (0.53, 3.42)	1.04 (0.38, 2.88)	1.34 (0.36, 4.85)	MEM				
1.02 (0.45, 2.25)	1.01 (0.46, 2.18)	0.85 (0.29, 2.4)	1.78 (0.9, 3.48)	1.3 (0.82, 2)	1.4 (0.68, 2.89)	0.77 (0.36, 1.59)	1.17 (0.83, 1.6)	1.24 (0.71, 2.11)	1.16 (0.6, 2.26)	1.39 (0.99, 1.94)	1.09 (0.71, 1.62)	1.39 (0.54, 3.45)	1.05 (0.38, 2.8)	MOF			
0.83 (0.38, 1.77)	0.82 (0.36, 1.84)	0.69 (0.24, 1.91)	1.45 (0.77, 2.71)	1.05 (0.71, 1.53)	1.14 (0.57, 2.22)	0.62 (0.29, 1.3)	0.95 (0.66, 1.32)	1.01 (0.62, 1.6)	0.94 (0.51, 1.75)	1.13 (0.85, 1.48)	0.89 (0.62, 1.24)	1.14 (0.45, 2.71)	0.85 (0.32, 2.21)	0.81 (0.57, 1.15)	PT		
1.14 (0.55, 2.36)	1.13 (0.5, 2.54)	0.94 (0.31, 2.78)	**1.98 (1.11, 3.63)**	1.44 (0.96, 2.22)	1.56 (0.83, 2.95)	0.85 (0.41, 1.77)	1.3 (0.93, 1.81)	1.38 (0.84, 2.32)	1.29 (0.66, 2.66)	**1.54 (1.03, 2.38)**	1.22 (0.94, 1.58)	1.56 (0.65, 3.67)	1.17 (0.42, 3.26)	1.11 (0.74, 1.74)	1.37 (0.94, 2.06)	TGC	
1.28 (0.35, 4.58)	1.26 (0.33, 4.66)	1.05 (0.61, 1.83)	2.23 (0.68, 7.59)	1.6 (0.54, 4.9)	1.75 (0.52, 6.04)	0.96 (0.27, 3.44)	1.44 (0.5, 4.26)	1.54 (0.5, 4.88)	1.45 (0.63, 3.46)	1.72 (0.64, 4.86)	1.36 (0.46, 4.07)	1.75 (0.44, 6.7)	1.3 (0.88, 1.97)	1.24 (0.43, 3.72)	1.52 (0.55, 4.48)	1.12 (0.37, 3.34)	TT_M

Note: Data are OR (95% CrI) of the row treatment relative to the column treatment in terms of clinical success rates. Bold values indicate comparisons that are statistically significant. ORs above 1 indicate higher rates of clinical success rates.

Supplementary league table S1C. The result of sensitivity analysis excluding studies with small sample size for clinical success rates

ALA																	
1.02 (0.36, 2.95)	AS																
1.25 (0.34, 4.36)	1.21 (0.33, 4.38)	CA_M															
0.58 (0.24, 1.37)	0.56 (0.22, 1.47)	0.47 (0.14, 1.54)	CEP_M														
0.8 (0.36, 1.72)	0.78 (0.33, 1.83)	0.63 (0.22, 1.91)	1.37 (0.72, 2.66)	CIP_M													
0.74 (0.3, 1.79)	0.72 (0.27, 1.92)	0.6 (0.18, 2.02)	1.27 (0.58, 2.79)	0.93 (0.47, 1.85)	CLI												
1.34 (0.5, 3.74)	1.31 (0.65, 2.66)	1.08 (0.31, 3.87)	2.32 (0.98, 5.73)	1.69 (0.78, 3.75)	1.81 (0.75, 4.56)	COT											
0.89 (0.41, 1.9)	0.86 (0.38, 1.96)	0.71 (0.25, 2.04)	1.52 (0.81, 2.94)	1.11 (0.75, 1.65)	1.2 (0.61, 2.35)	0.66 (0.31, 1.4)	CTX_M										
0.83 (0.36, 1.88)	0.81 (0.33, 1.97)	0.67 (0.22, 2.06)	1.43 (0.71, 2.95)	1.04 (0.6, 1.81)	1.12 (0.54, 2.35)	0.62 (0.27, 1.39)	0.94 (0.55, 1.61)	CUX_M									
0.9 (0.33, 2.27)	0.87 (0.32, 2.39)	0.72 (0.31, 1.63)	1.54 (0.64, 3.69)	1.12 (0.55, 2.26)	1.21 (0.49, 2.9)	0.66 (0.25, 1.69)	1.01 (0.51, 1.94)	1.08 (0.49, 2.28)	ERA								
0.75 (0.33, 1.61)	0.72 (0.31, 1.68)	0.6 (0.22, 1.61)	1.28 (0.66, 2.49)	0.94 (0.6, 1.44)	1.01 (0.5, 2)	0.55 (0.25, 1.19)	0.85 (0.58, 1.17)	0.9 (0.52, 1.51)	0.84 (0.48, 1.46)	ERM							
0.94 (0.47, 1.87)	0.92 (0.42, 2.05)	0.76 (0.26, 2.25)	1.63 (0.96, 2.8)	1.19 (0.81, 1.75)	1.28 (0.73, 2.28)	0.7 (0.34, 1.39)	1.07 (0.75, 1.52)	1.14 (0.72, 1.81)	1.06 (0.54, 2.14)	1.26 (0.86, 1.92)	IC						
0.74 (0.26, 2.18)	0.72 (0.23, 2.31)	0.6 (0.15, 2.37)	1.29 (0.48, 3.44)	0.93 (0.38, 2.37)	1.01 (0.37, 2.78)	0.55 (0.19, 1.62)	0.84 (0.35, 2.08)	0.89 (0.35, 2.36)	0.83 (0.29, 2.51)	0.99 (0.4, 2.55)	0.79 (0.35, 1.82)	ICRB					
1 (0.3, 3.31)	0.98 (0.28, 3.39)	0.81 (0.55, 1.18)	1.74 (0.56, 5.39)	1.27 (0.45, 3.45)	1.36 (0.42, 4.26)	0.75 (0.22, 2.44)	1.14 (0.42, 3.01)	1.21 (0.41, 3.48)	1.13 (0.54, 2.41)	1.35 (0.54, 3.38)	1.06 (0.38, 2.86)	1.36 (0.36, 4.93)	MEM				
1.02 (0.45, 2.24)	0.99 (0.45, 2.23)	0.82 (0.29, 2.35)	1.76 (0.9, 3.45)	1.28 (0.81, 2.01)	1.39 (0.68, 2.78)	0.76 (0.35, 1.6)	1.16 (0.82, 1.59)	1.23 (0.69, 2.12)	1.15 (0.58, 2.22)	1.37 (0.96, 1.94)	1.09 (0.71, 1.61)	1.37 (0.54, 3.4)	1.02 (0.38, 2.73)	MOF			
0.84 (0.38, 1.77)	0.81 (0.35, 1.84)	0.67 (0.24, 1.89)	1.44 (0.76, 2.73)	1.05 (0.7, 1.54)	1.13 (0.57, 2.19)	0.62 (0.28, 1.29)	0.95 (0.65, 1.32)	1.01 (0.62, 1.6)	0.94 (0.5, 1.76)	1.12 (0.84, 1.48)	0.89 (0.61, 1.24)	1.13 (0.45, 2.7)	0.83 (0.32, 2.18)	0.82 (0.57, 1.16)	PT		
1.15 (0.55, 2.4)	1.12 (0.5, 2.55)	0.92 (0.32, 2.76)	**1.98 (1.11, 3.65)**	1.44 (0.96, 2.22)	1.56 (0.84, 2.93)	0.86 (0.4, 1.77)	1.3 (0.94, 1.83)	1.38 (0.84, 2.33)	1.29 (0.66, 2.62)	**1.54 (1.04, 2.39)**	1.22 (0.94, 1.59)	1.55 (0.64, 3.62)	1.14 (0.42, 3.2)	1.12 (0.75, 1.74)	1.37 (0.96, 2.07)	TGC	
1.31 (0.37, 4.66)	1.28 (0.35, 4.72)	1.06 (0.61, 1.85)	2.27 (0.68, 7.53)	1.65 (0.55, 4.92)	1.79 (0.52, 5.96)	0.98 (0.27, 3.4)	1.49 (0.52, 4.31)	1.57 (0.51, 4.89)	1.46 (0.64, 3.52)	1.75 (0.65, 4.85)	1.4 (0.47, 4.04)	1.76 (0.44, 6.79)	1.31 (0.88, 1.97)	1.29 (0.45, 3.75)	1.57 (0.56, 4.49)	1.15 (0.38, 3.35)	TT_M

Note: Data are OR (95% CrI) of the row treatment relative to the column treatment in terms of clinical success rates. Bold values indicate comparisons that are statistically significant. ORs above 1 indicate higher rates of clinical success rates.

Supplementary league table S1D. The result of sensitivity analysis excluding studies with lack information of APACHE II score for clinical success rates

ALA																	
1.78 (0.47, 7)	CA_M																
0.57 (0.2, 1.56)	0.32 (0.09, 1.07)	CEP_M															
0.8 (0.3, 2.04)	0.45 (0.14, 1.3)	1.4 (0.63, 3.11)	CIP_M														
0.74 (0.25, 2.2)	0.41 (0.11, 1.48)	1.29 (0.51, 3.45)	0.92 (0.39, 2.32)	CLI													
1 (0.3, 3.42)	0.56 (0.14, 2.27)	1.77 (0.6, 5.4)	1.26 (0.47, 3.68)	1.36 (0.44, 4.35)	COT												
1.8 (0.33, 9.15)	1 (0.3, 3.22)	3.12 (0.64, 14.94)	2.24 (0.5, 9.61)	2.43 (0.46, 11.84)	1.77 (0.31, 9.42)	COX_M											
0.92 (0.35, 2.34)	0.51 (0.17, 1.42)	1.6 (0.74, 3.56)	1.15 (0.71, 1.92)	1.25 (0.52, 2.9)	0.91 (0.32, 2.46)	0.51 (0.13, 2.21)	CTX_M										
0.7 (0.23, 2.11)	0.39 (0.1, 1.42)	1.22 (0.48, 3.29)	0.88 (0.37, 2.22)	0.95 (0.34, 2.65)	0.69 (0.22, 2.2)	0.39 (0.08, 2.09)	0.76 (0.32, 1.86)	CUX_M									
1.05 (0.33, 3.25)	0.59 (0.23, 1.4)	1.84 (0.68, 5.05)	1.31 (0.6, 2.99)	1.43 (0.48, 4.05)	1.04 (0.31, 3.37)	0.59 (0.16, 2.27)	1.14 (0.56, 2.35)	1.5 (0.5, 4.34)	ERA								
0.79 (0.28, 2.08)	0.45 (0.15, 1.17)	1.4 (0.59, 3.17)	1 (0.56, 1.73)	1.08 (0.41, 2.57)	0.79 (0.26, 2.18)	0.44 (0.11, 1.84)	0.87 (0.55, 1.29)	1.14 (0.43, 2.75)	0.76 (0.4, 1.36)	ERM							
0.94 (0.41, 2.11)	0.53 (0.18, 1.52)	1.65 (0.91, 3.15)	1.17 (0.73, 2.03)	1.28 (0.62, 2.62)	0.93 (0.38, 2.27)	0.53 (0.13, 2.32)	1.02 (0.65, 1.67)	1.34 (0.64, 2.8)	0.9 (0.41, 2)	1.18 (0.7, 2.18)	IC						
0.73 (0.21, 2.57)	0.42 (0.1, 1.72)	1.29 (0.43, 4.1)	0.92 (0.33, 2.78)	1 (0.31, 3.27)	0.73 (0.2, 2.67)	0.41 (0.08, 2.46)	0.8 (0.29, 2.35)	1.04 (0.33, 3.48)	0.7 (0.21, 2.45)	0.92 (0.32, 2.9)	0.78 (0.31, 2.01)	ICRB					
1.43 (0.41, 5.12)	0.8 (0.49, 1.28)	2.51 (0.82, 8.15)	1.79 (0.68, 5.06)	1.93 (0.59, 6.52)	1.42 (0.38, 5.33)	0.8 (0.27, 2.45)	1.55 (0.62, 4.05)	2.02 (0.61, 6.95)	1.35 (0.65, 3)	1.79 (0.76, 4.64)	1.52 (0.59, 4.02)	1.93 (0.5, 7.54)	MEM				
1.1 (0.39, 2.9)	0.62 (0.2, 1.73)	1.93 (0.81, 4.45)	1.38 (0.76, 2.44)	1.5 (0.56, 3.62)	1.09 (0.36, 3.06)	0.61 (0.15, 2.68)	1.2 (0.77, 1.78)	1.57 (0.59, 3.86)	1.05 (0.49, 2.15)	1.38 (0.89, 2.17)	1.18 (0.62, 2.02)	1.5 (0.47, 4.3)	0.77 (0.28, 1.95)	MOF			
0.82 (0.29, 2.09)	0.46 (0.14, 1.27)	1.43 (0.61, 3.18)	1.03 (0.59, 1.67)	1.12 (0.41, 2.59)	0.81 (0.27, 2.21)	0.46 (0.11, 1.95)	0.9 (0.52, 1.38)	1.17 (0.43, 2.77)	0.78 (0.36, 1.55)	1.03 (0.66, 1.53)	0.87 (0.47, 1.45)	1.11 (0.35, 3.15)	0.57 (0.2, 1.42)	0.75 (0.45, 1.17)	PT		
1.14 (0.47, 2.75)	0.64 (0.22, 1.81)	**2 (1.00, 4.16)**	1.42 (0.86, 2.53)	1.55 (0.69, 3.42)	1.13 (0.43, 2.92)	0.64 (0.16, 2.78)	1.24 (0.82, 1.93)	1.63 (0.72, 3.66)	1.09 (0.51, 2.37)	1.43 (0.87, 2.58)	1.21 (0.86, 1.71)	1.56 (0.56, 4.12)	0.8 (0.31, 2.01)	1.03 (0.61, 1.9)	1.39 (0.84, 2.6)	TGC	
1.79 (0.45, 7.57)	1 (0.47, 2.2)	3.14 (0.89, 12.18)	2.24 (0.72, 7.72)	2.42 (0.65, 9.65)	1.78 (0.42, 7.8)	1 (0.3, 3.64)	1.95 (0.67, 6.22)	2.54 (0.67, 10.39)	1.7 (0.67, 4.79)	2.24 (0.8, 7.18)	1.9 (0.63, 6.19)	2.41 (0.56, 10.87)	1.25 (0.7, 2.37)	1.62 (0.55, 5.45)	2.17 (0.75, 7.56)	1.57 (0.53, 5)	TT_M

Note: Data are OR (95% CrI) of the row treatment relative to the column treatment in terms of clinical success rates. Bold values indicate comparisons that are statistically significant. ORs above 1 indicate higher rates of clinical success rates.

Supplementary league table S2A. The result of sensitivity analysis excluding studies with high-risk bias of blinding for microbiological success rates

CEP_M									
1.48 (0.44, 4.86)	CIP_M								
1.75 (0.42, 7.18)	1.18 (0.56, 2.5)	CTX_M							
2.02 (0.25, 17.54)	1.34 (0.26, 8.71)	1.12 (0.24, 6.96)	ERA						
1.67 (0.39, 7.21)	1.13 (0.5, 2.62)	0.96 (0.5, 1.86)	0.85 (0.16, 3.38)	ERM					
1.45 (0.79, 2.7)	0.98 (0.36, 2.73)	0.84 (0.23, 2.98)	0.72 (0.09, 5.09)	0.87 (0.23, 3.21)	IC				
1.19 (0.22, 7.92)	0.81 (0.12, 6.43)	0.7 (0.09, 6.08)	0.61 (0.04, 8.06)	0.72 (0.09, 6.71)	0.82 (0.17, 4.93)	ICRB			
2.22 (0.55, 9.46)	1.49 (0.71, 3.38)	1.28 (0.75, 2.27)	1.14 (0.2, 5.07)	1.33 (0.78, 2.37)	1.53 (0.44, 5.6)	1.84 (0.21, 14.87)	MOF		
1.85 (0.46, 7.44)	1.25 (0.63, 2.54)	1.06 (0.53, 2.12)	0.94 (0.16, 4.3)	1.11 (0.59, 2.08)	1.27 (0.37, 4.36)	1.54 (0.17, 11.8)	0.84 (0.45, 1.47)	PT	
1.71 (0.81, 3.93)	1.16 (0.39, 3.71)	0.99 (0.26, 3.96)	0.85 (0.1, 6.61)	1.02 (0.26, 4.3)	1.18 (0.74, 2.02)	1.44 (0.23, 7.71)	0.77 (0.2, 3.08)	0.92 (0.25, 3.61)	TGC

Note: Data are OR (95% CrI) of the row treatment relative to the column treatment in terms of microbiological success rates. ORs above 1 indicate higher rates of microbiological success rates.

Supplementary league table S2B. The result of sensitivity analysis excluding studies with small sample size for microbiological success rates

CEP_M										
1.52 (0.57, 4.25)	CIP_M									
1.97 (0.76, 5.9)	1.3 (0.65, 2.82)	CTX_M								
1.48 (0.54, 4.36)	0.98 (0.38, 2.58)	0.75 (0.28, 1.89)	CUX_M							
2.33 (0.38, 19.6)	1.53 (0.28, 11.09)	1.17 (0.22, 7.81)	1.57 (0.27, 11.99)	ERA						
1.95 (0.7, 6.51)	1.28 (0.58, 3.23)	0.99 (0.49, 2.09)	1.31 (0.5, 3.79)	0.85 (0.15, 3.74)	ERM					
1.46 (0.74, 2.92)	0.97 (0.45, 1.97)	0.74 (0.33, 1.49)	0.99 (0.43, 2.09)	0.63 (0.08, 3.39)	0.75 (0.29, 1.65)	IC				
1.2 (0.2, 8.13)	0.79 (0.13, 5.47)	0.61 (0.1, 4)	0.81 (0.13, 5.6)	0.51 (0.04, 6.04)	0.61 (0.09, 4.22)	0.82 (0.16, 4.91)	ICRB			
2.43 (0.91, 7.88)	1.6 (0.76, 3.85)	1.24 (0.7, 2.29)	1.64 (0.66, 4.63)	1.06 (0.17, 5.34)	1.25 (0.67, 2.36)	1.67 (0.8, 4.15)	2.05 (0.3, 13.09)	MOF		
1.75 (0.69, 4.75)	1.15 (0.58, 2.29)	0.89 (0.43, 1.69)	1.19 (0.52, 2.64)	0.76 (0.11, 3.72)	0.9 (0.44, 1.61)	1.2 (0.61, 2.43)	1.46 (0.22, 8.67)	0.72 (0.35, 1.26)	PT	
1.88 (0.88, 4.92)	1.25 (0.58, 3)	0.96 (0.46, 2.05)	1.28 (0.55, 3.31)	0.82 (0.12, 4.66)	0.98 (0.39, 2.37)	1.29 (0.84, 2.39)	1.59 (0.25, 9.06)	0.77 (0.33, 1.78)	1.08 (0.53, 2.48)	TGC

Note: Data are OR (95% CrI) of the row treatment relative to the column treatment in terms of microbiological success rates. ORs above 1 indicate higher rates of microbiological success rates.

Supplementary league table S2C. The result of sensitivity analysis excluding studies with lack information of APACHE II score for microbiological success rates

CEP_M										
1.68 (0.64, 4.66)	CIP_M									
2.08 (0.81, 5.86)	1.24 (0.67, 2.45)	CTX_M								
1.01 (0.35, 2.94)	0.6 (0.19, 1.9)	0.49 (0.15, 1.51)	CUX_M							
2.18 (0.37, 16.58)	1.29 (0.27, 8.21)	1.04 (0.23, 6.05)	2.18 (0.33, 17.5)	ERA						
1.89 (0.64, 5.94)	1.12 (0.54, 2.45)	0.91 (0.49, 1.6)	1.86 (0.54, 6.75)	0.88 (0.16, 3.43)	ERM					
1.45 (0.8, 2.7)	0.87 (0.4, 1.88)	0.7 (0.31, 1.49)	1.43 (0.61, 3.46)	0.67 (0.1, 3.45)	0.77 (0.3, 1.92)	IC				
1.24 (0.22, 8.01)	0.74 (0.12, 5.07)	0.59 (0.1, 4)	1.23 (0.2, 8.73)	0.57 (0.05, 6.42)	0.66 (0.1, 4.71)	0.85 (0.17, 5.02)	ICRB			
2.58 (0.92, 8.07)	1.54 (0.78, 3.33)	1.24 (0.75, 2.09)	2.54 (0.77, 9.35)	1.21 (0.21, 5.23)	1.36 (0.83, 2.41)	1.77 (0.76, 4.55)	2.1 (0.3, 13.59)	MOF		
2.11 (0.73, 6.56)	1.26 (0.67, 2.47)	1.02 (0.53, 1.92)	2.09 (0.62, 7.45)	0.98 (0.16, 4.46)	1.13 (0.61, 2.07)	1.46 (0.6, 3.68)	1.71 (0.24, 11.25)	0.82 (0.45, 1.43)	PT	
1.86 (0.94, 4.33)	1.11 (0.52, 2.59)	0.89 (0.44, 1.92)	1.83 (0.74, 5.29)	0.87 (0.13, 4.57)	0.99 (0.41, 2.52)	1.28 (0.86, 2.18)	1.52 (0.25, 8.38)	0.72 (0.31, 1.72)	0.88 (0.37, 2.22)	TGC

Note: Data are OR (95% CrI) of the row treatment relative to the column treatment in terms of microbiological success rates. ORs above 1 indicate higher rates of microbiological success rates.

Supplementary league table S3A. The result of sensitivity analysis excluding studies with lack information of APACHE II score for all-cause mortality

CA_M															
6.88 (0.52, 84.39)	CEP_M														
2.2 (0.21, 20.59)	0.33 (0.07, 1.39)	CIP_M													
1.19 (0.09, 14.77)	0.18 (0.03, 1.08)	0.54 (0.11, 2.49)	CLI												
2.23 (0.09, 55.36)	0.33 (0.02, 5.17)	1.01 (0.08, 14.53)	1.87 (0.14, 28.98)	COT											
1.18 (0.31, 4.89)	0.18 (0.01, 2.69)	0.54 (0.05, 6.41)	1 (0.07, 15.02)	0.54 (0.02, 14.35)	COX_M										
2.32 (0.23, 21.83)	0.35 (0.07, 1.56)	1.08 (0.54, 2.18)	1.98 (0.42, 9.7)	1.06 (0.07, 13.49)	1.96 (0.17, 21.63)	CTX_M									
3.44 (0.29, 35.88)	0.51 (0.1, 2.36)	1.54 (0.47, 5.59)	2.86 (0.58, 15.16)	1.54 (0.11, 20.14)	2.84 (0.22, 35.32)	1.43 (0.4, 5.18)	CUX_M								
3.97 (0.25, 56.83)	0.59 (0.06, 4.77)	1.87 (0.35, 9.03)	3.45 (0.38, 28.65)	1.76 (0.09, 35.34)	3.39 (0.19, 52.83)	1.72 (0.33, 7.92)	1.18 (0.16, 8.38)	ERA							
3 (0.26, 30.98)	0.46 (0.08, 2.44)	1.4 (0.51, 3.62)	2.6 (0.45, 14.17)	1.37 (0.09, 19.25)	2.54 (0.2, 30.25)	1.31 (0.52, 3)	0.92 (0.19, 3.93)	0.75 (0.21, 2.95)	ERM						
2.07 (0.21, 17.6)	0.31 (0.08, 1.07)	0.94 (0.43, 2.2)	1.74 (0.48, 6.69)	0.94 (0.08, 9.92)	1.72 (0.17, 17.25)	0.88 (0.38, 2.06)	0.61 (0.24, 1.53)	0.51 (0.09, 3.04)	0.67 (0.23, 2.23)	IC					
1.69 (0.73, 4.26)	0.25 (0.02, 2.89)	0.77 (0.1, 7.08)	1.42 (0.13, 17.24)	0.77 (0.03, 17.85)	1.42 (0.5, 4.05)	0.73 (0.09, 6.3)	0.5 (0.05, 5.03)	0.42 (0.03, 6.05)	0.56 (0.06, 5.41)	0.82 (0.11, 6.55)	MEM				
2.27 (0.21, 22.81)	0.34 (0.06, 1.69)	1.04 (0.44, 2.51)	1.91 (0.36, 10.11)	1.03 (0.07, 13.94)	1.92 (0.16, 22.54)	0.96 (0.46, 1.99)	0.68 (0.16, 2.67)	0.56 (0.13, 2.71)	0.73 (0.38, 1.59)	1.11 (0.38, 3.08)	1.35 (0.14, 11.4)	MOF			
2.35 (0.21, 23.26)	0.35 (0.06, 1.75)	1.08 (0.46, 2.37)	1.98 (0.38, 10.64)	1.06 (0.07, 14.46)	1.96 (0.16, 22.99)	1 (0.41, 2.27)	0.7 (0.16, 2.75)	0.58 (0.13, 2.8)	0.77 (0.35, 1.75)	1.14 (0.38, 3.17)	1.39 (0.15, 12.1)	1.03 (0.47, 2.26)	PT		
1.42 (0.16, 11.18)	**0.21 (0.05, 0.85)**	0.66 (0.28, 1.57)	1.2 (0.29, 5.2)	0.65 (0.05, 7.43)	1.19 (0.12, 10.88)	0.61 (0.27, 1.35)	0.42 (0.14, 1.31)	0.35 (0.07, 2.09)	0.47 (0.16, 1.49)	0.69 (0.37, 1.26)	0.84 (0.11, 5.75)	0.63 (0.22, 1.8)	0.61 (0.21, 1.84)	TGC	
0.98 (0.24, 3.57)	0.14 (0.01, 2.21)	0.44 (0.04, 5.44)	0.81 (0.06, 13.37)	0.43 (0.02, 11.77)	0.82 (0.18, 3.44)	0.41 (0.04, 4.86)	0.28 (0.02, 3.72)	0.24 (0.02, 4.18)	0.32 (0.03, 4.1)	0.46 (0.05, 4.94)	0.58 (0.19, 1.54)	0.42 (0.04, 5.39)	0.41 (0.04, 5.31)	0.67 (0.08, 6.63)	TT_M

Note: Data are OR (95% CrI) of the row treatment relative to the column treatment in terms of all-cause mortality. Bold values indicate comparisons that are statistically significant. ORs above 1 indicate higher rates of all-cause mortality.

Supplementary league table S4A. The result of sensitivity analysis excluding studies with lack information of APACHE II score for discontinuation due to adverse events

CA_M														
0.93 (0.08, 10.95)	CIP_M													
1.02 (0.05, 20.54)	1.07 (0.14, 8.75)	CLI												
0 (0, 1.86)	0 (0, 2.57)	0 (0, 2.88)	COX_M											
0.69 (0.06, 7.88)	0.73 (0.27, 1.87)	0.68 (0.1, 3.97)	0 (0, 3.52)	CTX_M										
0.79 (0.05, 12.06)	0.84 (0.18, 3.76)	0.78 (0.12, 4.69)	0 (0, 3.22)	1.17 (0.35, 3.7)	CUX_M									
2.09 (0.34, 13.19)	2.19 (0.41, 11.83)	2.02 (0.18, 24.77)	**0 (0, 0.91)**	3 (0.58, 17.09)	2.59 (0.35, 21.24)	ERA								
0.8 (0.09, 6.94)	0.83 (0.28, 2.51)	0.77 (0.09, 6.21)	0 (0, 2.92)	1.15 (0.38, 3.48)	0.98 (0.2, 4.89)	0.38 (0.1, 1.34)	ERM							
0.59 (0.05, 7.82)	0.63 (0.18, 2.15)	0.59 (0.11, 2.83)	0 (0, 4.17)	0.87 (0.39, 1.9)	0.75 (0.31, 1.78)	0.29 (0.04, 1.78)	0.76 (0.19, 2.89)	IC						
0.56 (0.03, 11.21)	0.6 (0.07, 4)	0.56 (0.05, 4.69)	0 (0, 5.09)	0.82 (0.13, 4.33)	0.7 (0.1, 3.92)	0.27 (0.02, 2.94)	0.72 (0.08, 5.25)	0.94 (0.17, 4.12)	ICRB					
2.09 (0.99, 4.51)	2.21 (0.21, 22.44)	2.04 (0.11, 39.16)	**0 (0, 0.86)**	3.05 (0.3, 32.11)	2.64 (0.2, 37.27)	1.01 (0.19, 5.23)	2.67 (0.34, 21.4)	3.53 (0.3, 42.62)	3.74 (0.21, 78.59)	MEM				
0.54 (0.05, 5.65)	0.57 (0.2, 1.48)	0.53 (0.06, 4.03)	0 (0, 4.39)	0.79 (0.28, 2.17)	0.68 (0.14, 3.15)	0.26 (0.06, 1.19)	0.69 (0.3, 1.59)	0.9 (0.24, 3.31)	0.96 (0.13, 8.18)	0.26 (0.03, 2.37)	MOF			
0.73 (0.07, 7.71)	0.77 (0.33, 1.77)	0.72 (0.09, 5.51)	0 (0, 3.14)	1.06 (0.39, 2.98)	0.91 (0.2, 4.33)	0.35 (0.08, 1.64)	0.94 (0.39, 2.24)	1.22 (0.34, 4.56)	1.29 (0.19, 11.24)	0.35 (0.04, 3.29)	1.35 (0.72, 2.69)	PT		
0.45 (0.04, 5.59)	0.47 (0.14, 1.47)	0.44 (0.07, 2.27)	0 (0, 5.49)	0.65 (0.34, 1.23)	0.56 (0.21, 1.51)	0.22 (0.03, 1.27)	0.57 (0.16, 1.98)	0.75 (0.47, 1.21)	0.79 (0.17, 4.65)	0.21 (0.02, 2.37)	0.83 (0.24, 2.8)	0.61 (0.18, 2.03)	TGC	
2.82 (0.43, 19.67)	3 (0.17, 54.28)	2.75 (0.09, 84.81)	**0 (0, 0.76)**	4.06 (0.24, 75.62)	3.55 (0.16, 82.39)	1.38 (0.13, 14.64)	3.6 (0.23, 54.67)	4.74 (0.24, 97.79)	5.08 (0.17, 168.18)	1.35 (0.23, 8.1)	5.21 (0.31, 87.45)	3.84 (0.24, 64.61)	6.29 (0.34, 127.97)	TT_M

Note: Data are OR (95% CrI) of the row treatment relative to the column treatment in terms of clinical success rates. Bold values indicate comparisons that are statistically significant. ORs above 1 indicate higher rates of discontinuation due to adverse events.

References

1. Lucasti C, Hershberger E, Miller B, Yankelev S, Steenbergen J, Friedland I, Solomkin J: **Multicenter, double-blind, randomized, phase II trial to assess the safety and efficacy of ceftolozane-tazobactam plus metronidazole compared with meropenem in adult patients with complicated intra-abdominal infections**. *Antimicrob Agents Chemother* 2014, **58**(9):5350-5357.
2. Solomkin J, Hershberger E, Miller B, Popejoy M, Friedland I, Steenbergen J, Yoon M, Collins S, Yuan G, Barie PS *et al*: **Ceftolozane/Tazobactam Plus Metronidazole for Complicated Intra-abdominal Infections in an Era of Multidrug Resistance: Results From a Randomized, Double-Blind, Phase 3 Trial (ASPECT-cIAI)**. *Clinical infectious diseases : an official publication of the Infectious Diseases Society of America* 2015, **60**(10):1462-1471.
3. Solomkin JS, Wilson SE, Christou NV, Rotstein OD, Dellinger EP, Bennion RS, Pak R, Tack K: **Results of a clinical trial of clinafloxacin versus imipenem/cilastatin for intraabdominal infections**. *Annals of surgery* 2001, **233**(1):79-87.
4. Solomkin J, Evans D, Slepavicius A, Lee P, Marsh A, Tsai L, Sutcliffe JA, Horn P: **Assessing the Efficacy and Safety of Eravacycline vs Ertapenem in Complicated Intra-abdominal Infections in the Investigating Gram-Negative Infections Treated With Eravacycline (IGNITE 1) Trial: a Randomized Clinical Trial**. *JAMA surgery* 2017, **152**(3):224-232.
5. Solomkin JS, Ramesh MK, Cesnauskas G, Novikovs N, Stefanova P, Sutcliffe JA, Walpole SM, Horn PT: **Phase 2, randomized, double-blind study of the efficacy and safety of two dose regimens of eravacycline versus ertapenem for adult community-acquired complicated intra-abdominal infections**. *Antimicrobial agents and chemotherapy* 2014, **58**(4):1847-1854.
6. Solomkin JS, Gardovskis J, Lawrence K, Montravers P, Sway A, Evans D, Tsai L: **IGNITE4: results of a Phase 3, Randomized, Multicenter, Prospective Trial of Eravacycline vs Meropenem in the Treatment of Complicated Intraabdominal Infections**. *Clinical infectious diseases* 2019, **69**(6):921-929.
7. Lucasti C, Vasile L, Sandesc D, Venskutonis D, McLeroth P, Lala M, Rizk ML, Brown ML, Losada MC, Pedley A *et al*: **Phase 2, Dose-Ranging Study of Relebactam with Imipenem-Cilastatin in Subjects with Complicated Intra-abdominal Infection**. *Antimicrob Agents Chemother* 2016, **60**(10):6234-6243.
8. Donahue PE, Smith DL, Yellin AE, Mintz SJ, Bur F, Luke DR: **Trovafloxacin in the treatment of intra-abdominal infections: results of a double-blind, multicenter comparison with imipenem/cilastatin. Trovafloxacin Surgical Group**. *American journal of surgery* 1998, **176**(6A Suppl):53S-61S.
9. Walker AP, Nichols RL, Wilson RF, Bivens BA, Trunkey DD, Edmiston CE, Jr., Smith JW, Condon RE: **Efficacy of a beta-lactamase inhibitor combination for serious intraabdominal infections**. *Ann Surg* 1993, **217**(2):115-121.
10. de Groot HG, Hustinx PA, Lampe AS, Oosterwijk WM: **Comparison of imipenem/cilastatin with the combination of aztreonam and clindamycin in the treatment of intra-abdominal infections**. *The Journal of antimicrobial chemotherapy* 1993, **32**(3):491-500.
11. Dela Pena AS, Asperger W, Kockerling F, Raz R, Kafka R, Warren B, Shivaprakash M, Vrijens F, Giezek H, DiNubile MJ *et al*: **Efficacy and Safety of Ertapenem Versus Piperacillin-Tazobactam for the Treatment of Intra-Abdominal Infections Requiring Surgical Intervention**. *Journal of Gastrointestinal Surgery* 2006, **10**(4):567-574.
12. Namias N, Solomkin JS, Jensen EH, Tomassini JE, Abramson MA: **Randomized, multicenter, double-blind study of efficacy, safety, and tolerability of intravenous ertapenem versus piperacillin/tazobactam in treatment of complicated intra-

abdominal infections in hospitalized adults. *Surgical infections* 2007, **8**(1):15-28.

13. Solomkin JS, Yellin AE, Rotstein OD, Christou NV, Dellinger EP, Tellado JM, Malafaia O, Fernandez A, Choe KA, Carides A *et al*: **Ertapenem versus piperacillin/tazobactam in the treatment of complicated intraabdominal infections: results of a double-blind, randomized comparative phase III trial**. *Annals of surgery* 2003, **237**(2):235-245.
14. Navarro Jr NS, Campos MI, Alvarado R, Quintero N, Branicki FJ, Wei J, Shivaprakash M, Vrijens F, Giezek H, Chan CY *et al*: **Ertapenem versus ceftriaxone and metronidazole as treatment for complicated intra-abdominal infections**. *International Journal of Surgery* 2005, **3**(1):25-34.
15. Yellin AE, Hassett JM, Fernandez A, Geib J, Adeyi B, Woods GL, Teppler H: **Ertapenem monotherapy versus combination therapy with ceftriaxone plus metronidazole for treatment of complicated intra-abdominal infections in adults**. *International journal of antimicrobial agents* 2002, **20**(3):165-173.
16. Yoshioka K, Youngs DJ, Keighley MR: **A randomised prospective controlled study of ciprofloxacin with metronidazole versus amoxicillin/clavulanic acid with metronidazole in the treatment of intra-abdominal infection**. *Infection* 1991, **19**(1):25-29.
17. Cohn SM, Lipsett PA, Buchman TG, Cheadle WG, Milsom JW, O'Marro S, Yellin AE, Jungerwirth S, Rochefort EV, Haverstock DC *et al*: **Comparison of intravenous/oral ciprofloxacin plus metronidazole versus piperacillin/tazobactam in the treatment of complicated intraabdominal infections**. *Annals of surgery* 2000, **232**(2):254-262.
18. Wacha H, Warren B, Bassaris H, Nikolaidis P: **Comparison of sequential intravenous/oral ciprofloxacin plus metronidazole with intravenous ceftriaxone plus metronidazole for treatment of complicated intra-abdominal infections**. *Surgical infections* 2006, **7**(4):341-354.
19. Solomkin JS, Reinhart HH, Dellinger EP, Bohnen JM, Rotstein OD, Vogel SB, Simms HH, Hill CS, Bjornson HS, Haverstock DC *et al*: **Results of a randomized trial comparing sequential intravenous/oral treatment with ciprofloxacin plus metronidazole to imipenem/cilastatin for intra-abdominal infections. The Intra-Abdominal Infection Study Group**. *Ann Surg* 1996, **223**(3):303-315.
20. Huizinga WK, Warren BL, Baker LW, Valleur P, Pezet DM, Hoogkamp-Korstanjep JA, Karran SJ: **Antibiotic monotherapy with meropenem in the surgical management of intra-abdominal infections**. *Journal of antimicrobial chemotherapy* 1995, **36 Suppl A**:179-189.
21. Kempf P, Bauernfeind A, Muller A, Blum J: **Meropenem monotherapy versus cefotaxime plus metronidazole combination treatment for serious intra-abdominal infections**. *Infection* 1996, **24**(6):473-479.
22. Chen CW, Ming CC, Ma CJ, Shan YS, Yeh YS, Wang JY: **Prospective, randomized, study of ampicillin-sulbactam versus moxifloxacin monotherapy for the treatment of community-acquired complicated intra-abdominal infections**. *Surgical infections* 2013, **14**(4):389-396.
23. De Waele J, Tellado J, Alder J, Reimnitz P, Jensen M, Hampel B, Arvis P: **Efficacy and safety of moxifloxacin vs. ertapenem in complicated intra-abdominal infections: Results of the PROMISE study**. *Clinical Microbiology and Infection* 2010, **16**(SUPPL. 2):S449.
24. Malangoni MA, Song J, Herrington J, Choudhri S, Pertel P: **Randomized controlled trial of moxifloxacin compared with piperacillin-tazobactam and amoxicillin-clavulanate for the treatment of complicated intra-abdominal infections**. *Annals of surgery* 2006, **244**(2):204-211.
25. Solomkin J, Zhao YP, Ma EL, Chen MJ, Hampel B: **Moxifloxacin is non-inferior to combination therapy with ceftriaxone plus metronidazole in patients with**

community-origin complicated intra-abdominal infections. *International journal of antimicrobial agents* 2009, **34**(5):439-445.

26. Weiss G, Reimnitz P, Hampel B, Muehlhofer E, Lippert H: **Moxifloxacin for the treatment of patients with complicated intra-abdominal infections (the AIDA Study)**. *Journal of chemotherapy (Florence, Italy)* 2009, **21**(2):170-180.
27. Ohlin B, Cederberg A, Forssell H, Solhaug JH, Tveit E: **Piperacillin/tazobactam compared with cefuroxime/ metronidazole in the treatment of intra-abdominal infections**. *The European journal of surgery = Acta chirurgica* 1999, **165**(9):875-884.
28. Brismar B, Malmborg AS, Tunevall G, Wretlind B, Bergman L, Mentzing LO, Nyström PO, Kihlström E, Bäckstrand B, Skau T: **Piperacillin-tazobactam versus imipenem-cilastatin for treatment of intra-abdominal infections**. *Antimicrobial agents and chemotherapy* 1992, **36**(12):2766-2773.
29. Erasmo AA, Crisostomo AC, Yan LN, Hong YS, Lee KU, Lo CM: **Randomized comparison of piperacillin/tazobactam versus imipenem/cilastatin in the treatment of patients with intra-abdominal infection**. *Asian journal of surgery / Asian Surgical Association* 2004, **27**(3):227-235.
30. Wang HJ, Xing XZ, Qu SN, Huang CL, Zhang H, Wang H, Yang QH, Yuan ZN: **A randomized controlled trial comparing the efficacy of tigecycline versus meropenem in the treatment of postoperative complicated intra-abdominal infections**. *Annals of palliative medicine* 2021, **10**(2):1262-1275.
31. Qvist N, Warren B, Leister-Tebbe H, Zito ET, Pedersen R, McGovern PC, Babinchak T: **Efficacy of tigecycline versus ceftriaxone plus metronidazole for the treatment of complicated intra-abdominal infections: results from a randomized, controlled trial**. *Surg Infect (Larchmt)* 2012, **13**(2):102-109.
32. Towfigh S, Pasternak J, Poirier A, Leister H, Babinchak T: **A multicentre, open-label, randomized comparative study of tigecycline versus ceftriaxone sodium plus metronidazole for the treatment of hospitalized subjects with complicated intra-abdominal infections**. *Clinical microbiology and infection* 2010, **16**(8):1274-1281.
33. Chen Z, Wu J, Zhang Y, Wei J, Leng X, Bi J, Li R, Yan L, Quan Z, Chen X *et al*: **Efficacy and safety of tigecycline monotherapy vs. imipenem/cilastatin in Chinese patients with complicated intra-abdominal infections: a randomized controlled trial**. *BMC infectious diseases* 2010, **10**:217.
34. Fomin P, Beuran M, Gradauskas A, Barauskas G, Datsenko A, Dartois N, Ellis-Grosse E, Loh E: **Tigecycline is efficacious in the treatment of complicated intra-abdominal infections**. *International Journal of Surgery* 2005, **3**(1):35-47.
35. Oliva ME, Rekha A, Yellin A, Pasternak J, Campos M, Rose GM, Babinchak T, Ellis-Grosse EJ, Loh E: **A multicenter trial of the efficacy and safety of tigecycline versus imipenem/cilastatin in patients with complicated intra-abdominal infections [Study ID Numbers: 3074A1-301-WW; ClinicalTrials.gov Identifier: NCT00081744]**. *BMC Infect Dis* 2005, **5**:88.
36. Chen Y, Zhu D, Zhang Y, Zhao Y, Chen G, Li P, Xu L, Yan P, Hickman MA, Xu X *et al*: **A multicenter, double-blind, randomized, comparison study of the efficacy and safety of tigecycline to imipenem/cilastatin to treat complicated intra-abdominal infections in hospitalized subjects in China**. *Ther Clin Risk Manag* 2018, **14**:2327-2339.
37. Barie PS, Vogel SB, Dellinger EP, Rotstein OD, Solomkin JS, Yang JY, Baumgartner TF: **A randomized, double-blind clinical trial comparing cefepime plus metronidazole with imipenem-cilastatin in the treatment of complicated intra-abdominal infections. Cefepime Intra-abdominal Infection Study Group**. *Archives of surgery (Chicago, Ill : 1960)* 1997, **132**(12):1294-1302.
38. Garbino J, Villiger P, Caviezel A, Matulionyte R, Uckay I, Morel P, Lew D: **A randomized prospective study of cefepime plus metronidazole with imipenem-cilastatin in the**

treatment of intra-abdominal infections. *Infection* 2007, **35**(3):161-166.

39. Lucasti C, Popescu I, Ramesh MK, Lipka J, Sable C: **Comparative study of the efficacy and safety of ceftazidime/avibactam plus metronidazole versus meropenem in the treatment of complicated intra-abdominal infections in hospitalized adults: results of a randomized, double-blind, Phase II trial**. *The Journal of antimicrobial chemotherapy* 2013, **68**(5):1183-1192.
40. Mazuski JE, Gasink LB, Armstrong J, Broadhurst H, Stone GG, Rank D, Llorens L, Newell P, Pachl J: **Efficacy and Safety of Ceftazidime-Avibactam Plus Metronidazole Versus Meropenem in the Treatment of Complicated Intra-abdominal Infection: results From a Randomized, Controlled, Double-Blind, Phase 3 Program**. *Clinical infectious diseases* 2016, **62**(11):1380-1389.
41. Qin X, Tran BG, Kim MJ, Wang L, Nguyen DA, Chen Q, Song J, Laud PJ, Stone GG, Chow JW: **A randomised, double-blind, phase 3 study comparing the efficacy and safety of ceftazidime/avibactam plus metronidazole versus meropenem for complicated intra-abdominal infections in hospitalised adults in Asia**. *International journal of antimicrobial agents* 2017, **49**(5):579-588.
42. Christou NV, Turgeon P, Wassef R, Rotstein O, Bohnen J, Potvin M: **Management of intra-abdominal infections. The case for intraoperative cultures and comprehensive broad-spectrum antibiotic coverage. The Canadian Intra-abdominal Infection Study Group**. *Arch Surg* 1996, **131**(11):1193-1201.
43. Angerås MH, Darle N, Hamnström K, Ekelund M, Engström L, Takala J, Viste A, Holme JB: **A comparison of imipenem/cilastatin with the combination of cefuroxime and metronidazole in the treatment of intra-abdominal infections**. *Scand J Infect Dis* 1996, **28**(5):513-518.

44. Merck Sharp & Dohme LLC. (2021). Ceftolozane/Tazobactam (MK-7625A) Plus Metronidazole Versus Meropenem for Participants with Complicated Intra-abdominal Infection. https://clinicaltrials.gov/ct2/show/NCT03830333?term=NCT03830333&draw=2&rank=1 [Accessed March 20,2021]
